# Comparative Configurational Process Analysis: A New Set-Theoretic Technique for Longitudinal Case Analysis

**DOI:** 10.1177/10944281241259075

**Published:** 2024-06-18

**Authors:** Christian Rupietta, Johannes Meuer

**Affiliations:** 1 Queen's Business School, 1596 Queen's University Belfast, Belfast, Northern Ireland, UK; 2 216541 Kühne Logistics University, Hamburg, Germany

**Keywords:** mixed methods design, qualitative comparative analysis, sequence analysis, process research, comparative longitudinal

## Abstract

In the past 20 years, researchers have significantly advanced various management fields by examining organizational phenomena through a configurational lens, including competitive strategies, corporate governance mechanisms, and innovation systems. Qualitative comparative analysis (QCA) has emerged as a primary method for empirically investigating organizational configurations. However, QCA has traditionally struggled to capture the temporal aspects of configurational phenomena. In this paper, we present configurational comparative process analysis (C^2^PA), which merges QCA with sequence analysis. We introduce the concept of configurational themes—recognizable temporal patterns of recurring combinations of explanatory conditions—to identify and track the temporal dynamics among these phenomena. We also outline configurational matching—a method for empirically identifying these themes by distinguishing theme-defining from theme-supporting conditions. C^2^PA allows researchers to explore the temporal dynamics of configurational phenomena, such as their stability, emergence, and decline at critical junctures. We illustrate the application of C^2^PA through a study of shareholder value orientation and discuss its potential for addressing key questions in management research.

## Introduction

How do configurational structures unfold over time? If corporate governance structures are conceptualized as bundles of practices, how consistently do these structures appear over time? If cognitive schemes emerge as patterns of beliefs, are these patterns subject to shocks or critical events? Organizational researchers are becoming increasingly interested in studying phenomena characterized by complex interdependencies among several causal or explanatory factors (Furnari et al., 2021; Slager et al., 2023). Common to such configurational research is conceptualizing cases from a set-theoretic perspective, accounting for conjunctural causation, equifinality, and asymmetric relationships (Misangyi et al., 2017). Researchers have adopted this perspective to study organizational phenomena, such as competitive strategies, corporate governance mechanisms, and national labor and innovation systems (Bell et al., 2014; Fiss, 2011; Gupta et al., 2020; Meuer et al., 2015).

The development of qualitative comparative analysis (QCA; Ragin, 2000) as the dominant method for configurational research has accompanied and accelerated interest in configurational research. To date, QCA has significantly contributed to various fields of organizational research. Researchers not only have successfully applied QCA to empirical research but also have made important methodological advancements in QCA (Duşa, 2018; Gabriel et al., 2018; Greckhamer et al., 2018; Meuer & Rupietta, 2017a; Rubinson et al., 2019). For example, researchers have increasingly conceptualized and analyzed configurational phenomena in terms of core and peripheral factors and the notion of neutral permutations—that is, the idea that “more than one constellation of different peripheral causes may surround the core causal condition” (Fiss, 2011, p. 398). Likewise, researchers are increasingly expanding the applicability of QCA to large-N settings and have started integrating and mixing QCA with other methods.

Despite these significant advancements, one of the most severe limitations of QCA relates to its limited ability to study the temporal dynamics of configurational phenomena. This limitation is problematic because it prevents researchers from exploring time-related aspects of configurational phenomena, such as temporal changes in the relative importance of explanatory factors or the emergence and decline of configurational phenomena. Yet, questions about the temporal dynamics of configurational phenomena are becoming increasingly important. Although researchers have proposed approaches for incorporating time (e.g., Caren & Panofsky, 2005; Hak et al., 2013; Pagliarin & Gerrits, 2020), it remains a challenge for QCA and configurational research to handle temporal dynamics and processes.

In this paper, we contribute to the emerging research on time and QCA by offering an innovative technique—comparative configurational process analysis (C^2^PA)—that integrates QCA with sequence analysis. Sequence analysis is a quantitative method for process research that emerged in the social sciences during the 1980s (Abbott, 1990; Abbott & Tsay, 2000; Langley et al., 2013; Liao et al., 2022). Organizational researchers have become increasingly interested in sequence analysis (Biemann & Datta, 2014; Venkatraman et al., 1994). Sequence analysis systematically identifies and compares multiple sequences of steps or events and in so doing allows researchers to study generalizable temporal patterns (Elzinga & Studer, 2015; Ritschard & Studer, 2018). Building on the unique strengths and common underlying analytical logics of QCA and sequence analysis, C^2^PA is designed to explore the temporal dynamics of configurational phenomena. It thereby offers new opportunities for researchers to identify similarities across configurational sequences, what we label “configurational themes” (i.e., recognizable temporal patterns of recurring combinations of explanatory conditions), and analyze the form and fit of these configurational themes to address aspects of their stability, emergence, and decline at different junctures.

To illustrate C^2^PA, we explored the temporal dynamics among shareholder value orientation (SVO) among 54 companies listed on the German HDAX, an index that includes the 110 largest equities in Prime Standard, between 2006 and 2017. This setting is useful for illustrating C^2^PA because research on SVO has conceptualized ownership structures as configurations of different shareholders and theorized about the importance of temporal dynamics internal and external to ownership configurations (e.g., when the objectives and the power of shareholders or societal views of SVO change; Fiss & Zajac, 2004; Meyer & Höllerer, 2010). Through a repeated cross-sectional QCA, we identify three temporally robust configurational themes of shareholders associated with a strong SVO. We subsequently translate the output of QCA into configurational panel data and use sequence analysis to explain the consequences of changes in ownership configurations for a company's SVO. Our illustration helps clarify the unique opportunities of C^2^PA and allows us to outline practical recommendations for researchers interested in applying C^2^PA to analyze the temporal dynamics of configurational themes.

We have structured this paper as follows. QCA and the challenges of handling temporal dynamics section discusses the challenges that QCA faces in addressing temporal dynamics and introduces sequence analysis as a complementary method to overcome these obstacles. Comparative configurational process analysis (C^2^PA) section provides an overview of the C^2^PA procedure, demonstrating its application through a case study of the adoption of a strong SVO among stock market-listed German companies from 2006 to 2017. Discussion section discusses the potential contributions of C^2^PA to the literature on time and QCA and to the broader literature on mixed-methods designs. Here, we also offer considerations for its use and suggest promising fields in the management literature for addressing substantive questions. Conclusion section concludes this work.

## QCA and the Challenges of Handling Temporal Dynamics

### A Brief History of QCA

QCA originated in the field of comparative international politics during the 1980s as a formalized method that uses set theory and Boolean algebra to systematically compare cases as configurations, defined as combinations of (causal) conditions (Ragin, 1987, 2008). QCA's systematic approach to comparing cases and its associated configurational perspective has offered researchers the opportunity to identify combinations of explanatory conditions as sufficient for an outcome to occur, build and test typology theories, and handle situations of considerable complex causation. QCA, for example, allows researchers to identify specific combinations of conditions associated with an outcome (known as “conjunctural causation”), determine if multiple combinations of conditions are associated with an outcome (known as “equifinality”), and demonstrate that the negation of a configuration does not necessarily explain the occurrence of the negation of the outcome (known as “causal asymmetry”).

In management research, QCA emerged during the 2010s, with several conceptual, methodological, and empirical studies originally applied to organization theory (Fiss, 2011; Greckhamer, 2011). Since then, QCA has been applied throughout the management research field. For example, Misangyi and Acharya (2014) contribute to governance research by using QCA to develop a new theory about how governance mechanisms for mitigating agency problems operate effectively as complements (mutually enhancing) or as substitutes (mutually exclusive). In international management, Greckhamer (2016) uses QCA to explore how formal and informal country-level institutions shape differences in executive compensation. Halme et al. (2020) uses QCA in the field of sustainability management to identify pathways of institutional pressures, the accountability of company ownership, and CSR implementation that lead to better environmental and social performance. Other studies using QCA have contributed to research on human resource management, entrepreneurship, organizational behavior and operations research, innovation policy and management, and international management (e.g., Arellano et al., 2021; Fainshmidt et al., 2020; Meuer, 2017; Nikou et al., 2022; Zimmermann et al., 2018).

Although QCA was created in the tradition of case-based comparative research, researchers have increasingly applied it to analyze larger samples (Greckhamer et al., 2013). Moreover, as a research approach inspired by case-study research that is also formalized and systematic, QCA has frequently been a component of mixed-methods approaches, integrating QCA with both qualitative and quantitative methods (Gabriel et al., 2018; Greckhamer et al., 2008; Meuer & Rupietta, 2017a, 2017b). Consequently, research and publications using QCA have grown rapidly over the past two decades. To date, this method has been used in studies published in all leading academic journals. Overall, organizational researchers across various fields have successfully used QCA to advance the scholarly understanding of configurational phenomena (such as team composition, governance systems, and labor market structures).

### QCA's Limited Ability to Account for Time

While the interest in and use of QCA has proliferated in management research, researchers increasingly highlight QCA's limited ability to incorporate aspects of time and processes into the analysis of configurational phenomena (e.g., Caren & Panofsky, 2005; Fischer & Maggetti, 2017). Indeed, for 30 years, researchers have attempted to incorporate time and processes into QCA (Caren & Panofsky, 2005; Schneider, 2019; Schneider & Rohlfing, 2013). For example, Ragin (1994) proposed using time-related conditions (e.g., an additional condition indicating the temporal order of events). Caren and Panofsky (2005) developed temporal QCA (TQCA), which involves predefining and specifying sequences of conditions (e.g., A must occur before B, etc.). More recently, Aversa et al. (2015) have used cross-sectional QCA (multiple QCAs, different time periods) that involves conducting and comparing several QCA analyses for each point in time (see also Nishant & Ravishankar, 2020 for a related application).

Other researchers have proposed adjusting QCA (e.g., the temporal use of the two-step QCA) or combining QCA with other methods, such as process tracing or panel data set-theoretic methods to address time-related questions with QCA (Beach & Rohlfing, 2018; Fischer & Maggetti, 2017; García Castro & Ariño Martín, 2013; Schneider & Rohlfing, 2013). Further, coincidence analysis (CNA), a method closely related to QCA, emphasizes the analysis of causal chains between conditions and thereby is capable to explain the occurrence of potential intermediary outcomes before explaining the final outcome (Baumgartner, 2013; Baumgartner & Epple, 2014). The diversity of approaches for advancing QCA according to time has also led to reviews and typologies summarizing and structuring approaches to time and QCA (Fischer & Maggetti, 2017; Pagliarin & Gerrits, 2020; Verweij & Vis, 2021).

Yet, for several reasons, despite these significant advancements, it remains challenging for empirical QCA research to handle temporal dynamics and processes. Incorporating time increases modeling challenges (e.g., when adding conditions to account for temporal causal aspects), requires additional upfront theorizing (e.g., the need to account for temporal sequences of conditions), and introduces more vagueness (e.g., at what stage during the research process should time be incorporated).

At the same time, questions about temporal dynamics, processes, and sequences of events or phases are central to organizational research (Abbott, 1990; Langley et al., 2013; Pentland et al., 2020). Early configurational theorizing already speculated about temporal dynamics of configurations, discussing the stability and prevalence of orchestrating themes, the forces that may disrupt the integrative mechanisms that explain the co-occurrence of attributes, and the dangers of organizations to adopt, for example, overly simple configurations (Furnari et al., 2021; Miller, 1987, 1996). Clearly, configurational phenomena also may be subject to complex causal dynamics. For example, if one conceptualizes governance mechanisms or HR practices as bundles (i.e., configurations), do these bundles evolve over time? How temporally stable are they? Do minor changes in the composition of conditions lead to a decline and reemergence among configurations? Do these trends influence the efficiency of the internal mechanism (fit) of configurations? To what extent do global or local exogenous shocks, such as changes in a legal system or environmental disasters, alter the structure of configurations? The inability of QCA to account for time remains problematic because it prevents researchers from addressing questions about how organizational configurations “evolve in form and substance over time” (Ketchen, 2013, p. 305).

### Introducing Sequence Analysis to Address Temporal Dynamics in QCA

In this paper, we present a new technique for integrating time into QCA, combining QCA with sequence analysis. Sequence analysis originated in biology during the late 1960s to analyze DNA-based genomes and diffused during the 1980s into the social sciences. Since then, it has increasingly been applied, especially in sociology, for example, for analyzing life courses (Aisenbrey & Fasang, 2010; Liao et al., 2022). Sequence analysis is a formalized method for studying processes, that is, research concerned with understanding concepts related to activities, sequences of change, and the temporal ordering of events or phases (Abbott, 1990; Langley et al., 2013; Liao et al., 2022; Ritschard & Studer, 2018). Unique to sequence analysis is its ability to systematically identify multiple sequences (trajectories or pathways). In so doing, it provides insights into more generalizable temporal patterns.

In management research, sequence analysis is rapidly gaining interest, for example, in studying how successful careers (e.g., those of Fortune 100 CEOs) unfold, unravel resource allocation activities in product development processes, and explain the emergence of market structures (Jeong & Leblebici, 2019; Koch et al., 2017; Koch et al., 2021; Storz, 2008). Today, sequence analysis is an established process research method with substantive methodological developments, such as visualization and model coefficients. It is particularly useful “for testing and developing theoretical arguments about the speed, order, and timing of processes” (Brzinsky-Fay & Kohler, 2010; Liao et al., 2022, p. 14).

Several techniques for the comparison of sequences exist, with optimal matching being the most commonly used (Brzinsky-Fay & Kohler, 2010). This technique involves four generic steps (e.g., Abbott & Tsay, 2000; Biemann & Datta, 2014; Brzinsky-Fay & Kohler, 2010). In Step 1, the researcher codes the data into temporally ordered data points that describe sequences of phases or events. In Step 2, one defines the indel and substitution costs—that is, how costly it would be to change one sequence to make it identical to another. In Step 3, one calculates the similarity (or dissimilarity) between sequences to assess how related (i.e., similar) the two sequences are. In Step 4, the researcher uses cluster analysis to identify groups of cases with similar sequences to elicit common (conceptual) pathways. While these four steps are generic for all sequence analyses, researchers have various modeling choices in each of the four steps.

Among the available techniques for generating dissimilarity measures, most researchers use optimal matching to assess the (dis-)similarity of sequences (Abbott & Tsay, 2000; Biemann & Datta, 2014). Optimal matching measures how many insertions, deletions, or substitutions of sequence elements one must perform to transform one sequence into a second one. In so doing, optimal matching identifies the longest partially matching sequence, also known as the “common backbone” or “common narrative” between trajectories (Elzinga & Studer, 2015). Researchers have criticized optimal matching for its atheoretical approach to substitution costs and for its potential overparameterization due to the many possible ways for researchers to specify indel and substitution costs. To address some of these criticisms, researchers have developed alternative variants of optimal matching that—depending on the researchers’ assumptions—are sensitive to the length of spells, sequences of transitions, or sequences of spells (Elzinga & Studer, 2015). These alternatives greatly increase the usefulness and applicability of sequence analysis for researchers interested in temporal dynamics, trajectories, and sequences of steps or events.

Modeling choices also exist for defining distance measures. Distance measures are a key analytical consideration in sequence analysis because they reflect the researcher's assumptions about the similarity (or dissimilarity) of sequences. For example, researchers may assume that sequences are similar if they share (1) the longest continuous spell (sequence duration), (2) a high number of matching subsequences (sequencing), or (3) a high number of switches between states (timing; Studer & Ritschard, 2016). A researcher may also make assumptions about how “costly” the substitution of two states may be and, thus, incorporate substantive or theoretical reasoning into substitution costs. Hence, depending on the researcher's interest, sequence analyses may incorporate different assumptions about trajectories’ timing, sequencing, or duration. Last, sequence analysis offers several approaches to clustering that differ significantly in their alternative clustering techniques, for example, Markov models, divisive property-based methods, and fuzzy clustering (Ritschard & Studer, 2018) as well as important alternative dissimilarity measures.

In summary, sequence analysis and optimal matching, as the most common technique, have significantly advanced over the past three decades and have received increased interest from organizational researchers (Andresen et al., 2015; Biemann & Datta, 2014). It offers a structured and formalized method for analyzing longitudinal data as sequences of elements. Sequence analysis and QCA also share several important similarities. For example, both methods draw on a systematic and formalized comparison of sequences and cases. Moreover, researchers point toward similar methodological developments, for example, calling for a “stronger interaction with related approaches” (Ritschard & Studer, 2018, p. 3) to use sequence analysis for case identification, improve the representativeness of its results, or identify situations in which multiple trajectories unfold jointly (Gauthier et al., 2010; Le Roux et al., 2020). While debates continue about QCA's challenges with temporal dynamics, similar debates are occurring in the literature on sequence analysis, for example, concerning questions of addressing equifinality (e.g., Borgna & Struffolino, 2018). Thus, we argue that the two methods offer complementary strengths that open new avenues for analyzing configurational phenomena in a formalized and structured way over time.

## C^2^PA

**Figure 1.**
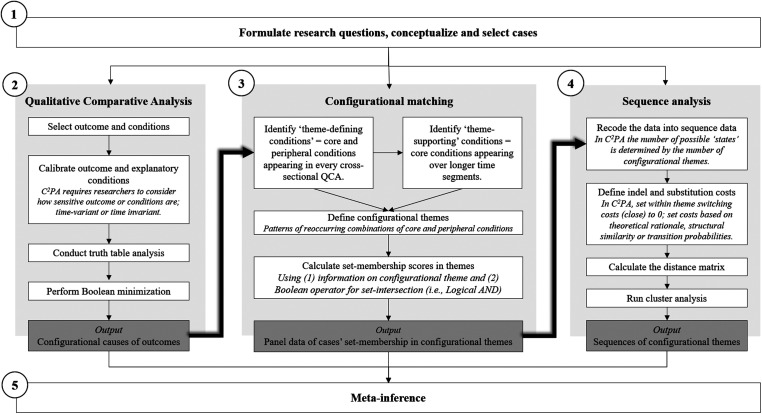
Flowchart for comparative configurational process analysis.

The technique we present in this paper—C^2^PA integrates QCA and sequence analysis in a sequential mixed-methods approach that consists of five steps. Figure 1 shows the flow chart of C^2^PA and includes the generic protocol for QCA and sequence analysis. Step 1 involves the formulation of a research question that focuses on the temporal dynamics at the case and theme levels over discrete points in time. This step requires the selection of cases for the analysis and conceptualization of the temporal dynamics of configurational themes. Step 2 consists of a repeated cross-sectional QCA (i.e., a QCA for each point in time). It follows the standard QCA protocol and helps identify configurations that lead to the occurrence of the outcome at each point in time.

Step 3 involves a process central to C^2^PA that we label “configurational matching.” Through this process, researchers identify recurring patterns in the QCA results and aggregate these patterns into configurational themes. In so doing, the process enables researchers to transform case-level dynamics between configurational themes into sequences. Step 4 comprises a sequence analysis following the standard sequence analysis protocol to analyze the case-level dynamism of configurational themes and to cluster similar temporal patterns across cases. Finally, Step 5 covers the interpretation of the theme and case-level dynamics. Together, these five steps make C^2^PA and allow researchers to integrate time and process perspectives into configurational research. In the following, we explain each step of the application of C^2^PA with an exemplary study on SVO (e.g., Carberry & Zajac, 2021; Fiss & Zajac, 2004; La Porta et al., 1999; Prahalad, 1994).

### Step 1: Theoretical Background, Research Questions, and Sample Selection

During the 1990s and 2000s, SVO, also labeled “shareholder primacy,” was hailed as the most advanced and soon-to-be-dominant governance form for capitalist organizations (Rappaport, 2006; Stout, 2012). In recent decades, research on financial economics and legal studies has investigated the emergence and consequences of SVO (Fligstein & Goldstein, 2015; Hansmann & Kraakman, 2017; Seo et al., 2016). Sociologists and political economists have explored the institutional environments conducive to (or impeding) the widespread adoption of SVO among national business systems and have evaluated the effect of its emergence on the behavior of financial markets and economic inequality (Rubach & Sebora, 1998). Organizational researchers have also shown interest in the SVO and have examined how ownership concentration and other socioeconomic conditions explain the adoption of a strong SVO, how this seminal governance form has diffused among companies, and how societies frame and make sense of the meaning of such an important concept (Blair, 2003; Fiss & Zajac, 2004; Hillman & Keim, 2001; Meyer & Höllerer, 2010). Shareholder value has received a great deal of attention from both industry and research in recent decades.

Research on SVO is useful for our purposes for two reasons. First, whereas research on SVO has traditionally examined the concentration of ownership as a function of the emergence of an SVO, the results have been inconclusive (Carberry & Zajac, 2021; Fiss & Zajac, 2004; Meyer & Höllerer, 2010). Researchers have argued that shareholders may vary in their objectives, powers, and interests. Ownership concentration, that is, the dominance of one particular type of shareholder with idiosyncratic interests and powers, may be insufficient for capturing the multitude of diverging interests among ownerships. Hence, rather than assessing the influence of single, dominating, concentrated types of owners, researchers have argued for important dynamics “across and within ownership categories” (Fiss & Zajac, 2004, p. 572) explaining the adoption of SVO and have called for a configurational perspective on ownership.

Second, although SVO has long been considered the only feasible and most dominant governance model, the idea of a primary shareholder has been increasingly questioned (Denning, 2017). The adoption of SVO, itself, may change over time with alterations in the ways (pathways) of how companies adopt SVO. Micropolitical dynamics internal to the company may cause these shifts in governance models, for example, when the objectives and power statuses of shareholders among each other may change over time (Fiss & Zajac, 2004; Meyer & Höllerer, 2010). Simultaneously, global or local exogenous events may fundamentally challenge the dominance of SVO. For example, temporary events (e.g., the COVID crisis or the war in Ukraine) may question a management approach targeting shareholders’ objectives. Likewise, macroeconomic trends, such as political and cultural changes in the corporate environment, may lead to shifts in governance models and changes in SVO over time. Correspondingly, both external and internal factors may cause temporal changes in the emergence or decline of SVO.

These insights from research on SVO also raise new research questions. Are there different ownership configurations associated with the adoption of SVO? How stable are these ownership configurations over time, and when do they emerge? Do they decline and reemerge, and are they subject to internal and external temporal dynamics? Do companies maintain a strong SVO even if they change their ownership configuration? Together, the call for conceptualizing SVO as configurations of ownership types and for a better understanding of exogenous triggers that cause temporal dynamics in firms’ SVO adoption makes this setting valuable for demonstrating the application and usefulness of C^2^PA.

To address these questions and illustrate C^2^PA, we compile and analyze the ownership structures among a balanced panel of all companies listed on the HDAX (balanced because we have data available on all companies in all years). The HDAX includes the 110 largest stocks traded on the Frankfurt Stock Exchange; its composition is revised annually as larger stocks are listed and smaller ones are delisted. We retrieve data on ownership shares from Thomson Reuters’ Refinitiv database, and after excluding cases with missing data, our final dataset contains 54 companies from 2006 through 2017. Our data include the largest stock market-traded companies in Germany, such as the Mercedes-Benz Group, BMW, and Volkswagen, as well as high-tech companies, such as SAP and Siemens. The ownership configurations of these 54 companies vary, and we observe companies during a period of economic and political turmoil (e.g., global economic crises and the Fukushima nuclear disaster). These data are appropriate for studying the ownership configurations and temporal dynamics of SVO.

### Step 2: Repeated Cross-Sectional QCA

In Step 2, to identify the ownership configurations among HDAX-listed companies between 2006 and 2017, we conduct a cross-sectional QCA following the generic QCA protocol: (1) selecting the outcome and conditions, (2) calibrating the outcome and the conditions by assigning membership scores based on substantive and theoretical knowledge, (3) analyzing the truth table, and (4) conducting Boolean minimization (Greckhamer et al., 2018; Ragin, 2008; Schneider & Wagemann, 2013). First, we defined the outcome and explanatory conditions for the QCA model. For our outcome, we follow earlier research on SVO and develop a composite scale of nine items that reflect strong SVO. Items on our scale include, for example, whether the company has a policy for shareholder engagement or for limiting shareholders’ rights to capital (reverse coded) and whether shareholders vote on executive positions. (Supplementary file A includes a technical note on the full list of items and descriptive statistics on our outcome variable measure, SVO.)

The explanatory conditions in our model reflect the ownership structure of a company. Drawing on La Porta et al. (1999), we group ownership into five shareholder groups: hedge funds, public investors (governments), profit-oriented organizations (corporations), holdings, and individual investors. These ownership types are associated with different interests and objectives regarding companies’ adoption of an SVO. Whereas hedge funds are widely considered shareholders likely to demand strong SVO from a company's management, public investors are usually viewed as adopting weaker SVO. Evidence for profit-oriented organizations is mixed because owners may pursue substantially different investment objectives characteristic of the collision of interests typical of different stakeholders (Fligstein & Goldstein, 2015; La Porta et al., 1999). Holding companies own interests in several other companies. However, unlike corporations, they are not involved in day-to-day operations. One reason why one may choose to register a holding company rather than a corporation lies in the way holding companies are taxed. In Germany, like many other countries, holdings enjoy tax benefits; they are exempt from paying taxes on dividends and capital gains. Thus, shareholders differ in their primary objectives for holding shares of a company and, accordingly, in their relative power status with respect to one another.

Second, we calibrate the outcome and the five conditions. Calibration uses anchor points based on theoretical or substantive knowledge to allocate a qualitative interpretation to a value and define the degree of cases’ set membership (Rubinson et al., 2019). To transform data into set membership scores ranging from 0 to 1, a researcher needs to define anchors that build on theoretical or substantive knowledge. In the context of C^2^PA, calibration deserves special attention for two reasons. QCA requires that each set is calibrated using a theoretical and substantive anchor; these anchors may be time sensitive or time insensitive, depending on the qualitative meaning of a set. Meanwhile, sequence analysis requires that each state be defined in the same way at each time point. Consequently, a consistent qualitative meaning of a set is essential for both QCA and sequence analysis.

We calibrate the outcome as the set of companies with above-average annual SVO. Because research has shown that companies adopt a stronger SVO over time, we calibrate SVO as a “time-sensitive” set to ensure that we measure important differences in kind. We anchor our calibration points in each year to the distribution of firms’ SVO across the entire population of companies listed on the HDAX. We define the 10th percentile as the threshold for being fully out of the set, the mean as the crossover point, and the 90th percentile as the threshold for being fully in the set. While our calibration may lead to minor differences in the measurement of states at each time point, it ensures that the definition of a state is conceptually identical across years.

To calibrate the conditions, we use “time-insensitive” anchor points that build on the voting rights defined by the German Stock Companies Act (AktG, 2023). Specifically, we define for each ownership type the set of companies with influential owners, setting the full nonmembership at 3% of shares (minority shareholder), the crossover point at 5% (minority shareholder with legal reporting obligations), and the full membership at 25% (minority shareholder with blocking rights). Table 1 provides an overview of the calibration approach for the outcome and explanatory conditions in our example.

**Table 1.** Calibration of Outcome and Explanatory Conditions.

	Measure	Calibration	Calibration anchors			Explanation
Outcome condition						
The set of companies with an above-average annual SVO	Composite scale of nine items	Time-sensitive	Fully-out: 10th percentile	Crossover: Mean	Fully-in: 90th percentile:	To calibrate the outcome, strong SVO, we use a relative benchmark in each year of the panel data set. The benchmarks come the SVO measure of all HDAX companies in the respective year, not only the companies in the balanced panel. The outcome is thus the set of companies with above average annual SVO (i.e., above-average SVO relative to the HDAX average).
Explanatory conditions						
The set of companies with influential hedge funds	Percentage of shares held by hedge funds	Time-insensitive	Fully-out: 3%	Crossover: 5%	Fully-in: 25%	To calibrate ownership shares, we use the information on voting rights defined by the German Stock Companies Act (Aktiengesetz). Specifically, we use three anchor points that are substantively important because they define the scope of shareholders’ reporting duties. We consider a percentage of shares below 3% as being “out” of the set because, at this threshold, shareholders are required to publicly report their ownership stakes in both the focal company and any ownership share above 3% in other companies. Moreover, we use a percentage of shares of 5% as the cross-over point, because at 5% the Aktiengesetz allows minority shareholders to call for an official shareholder meeting. Finally, we consider a percentage of shares above 25% as being fully “in” the set because, beyond this threshold shareholders hold a “blocking minority” that allows them to prevent certain resolutions and thus significantly influence a company's orientation. **1**. Two changes: Please amend text to say Stock Corporation Act.
The set of companies with influential governmental investors	Percentage of shares held by government entities	Time-insensitive	Fully-out: 3%	Crossover: 5%	Fully-in: 25%	
The set of companies with influential corporate investors	Percentage of shares held by profit-oriented organizations	Time-insensitive	Fully-out: 3%	Crossover: 5%	Fully-in: 25%	
The set of companies with influential holdings investors	Percentage of shares held by holdings	Time-insensitive	Fully-out: 3%	Crossover: 5%	Fully-in: 25%	
The set of companies with influential individual investors	Percentage of shares held by individual investors	Time-insensitive	Fully-out: 3%	Crossover: 5%	Fully-in: 25%	

*Note*. SVO = shareholder value orientation.

Third, QCA proceeds with the truth table analysis. A truth table contains all logically possible combinations of present and absent conditions and links these combinations to the outcome. We used fs/QCA 3.0 (Ragin & Davey, 2014) to generate a truth table for each year of our data set. Every truth table provides information regarding the raw consistency, the proportional reduction in inconsistency (PRI), and the frequency of each configuration.^1^ We use this information to select those configurations for the minimization and set the consistency cutoff at 0.80, the PRI consistency cutoff at 0.65, and the frequency cutoff at 1 for all years. (Supplementary file B includes an aggregated truth table.) Fourth, we perform the Boolean minimization through which one minimizes a truth table. For the intermediate solution, we define two directional expectations. Following research on different shareholder investment objectives, we assume that companies will adopt strong SVO either in the presence of the strong ownership of hedge funds or in the absence of strong government ownership.

**Figure 2.**
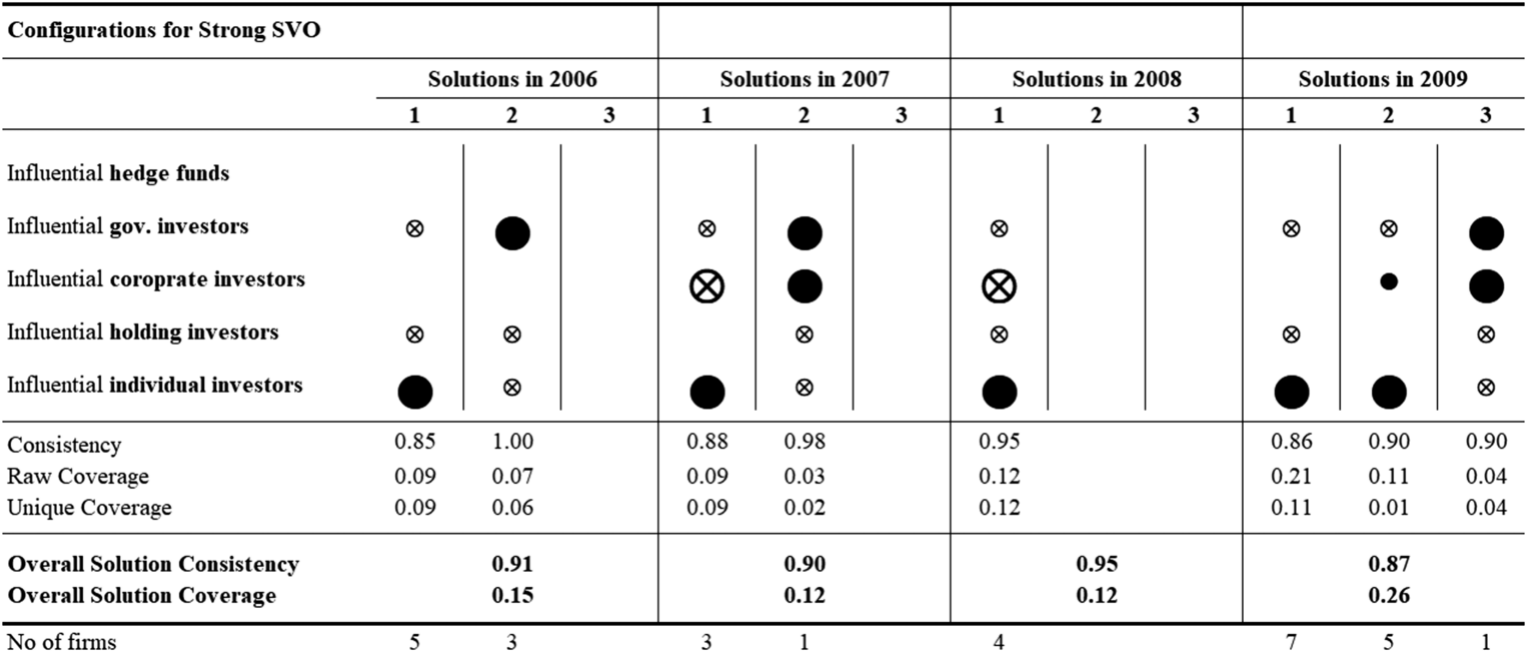
Configurational charts 2006–2009.

**Figure 3.**
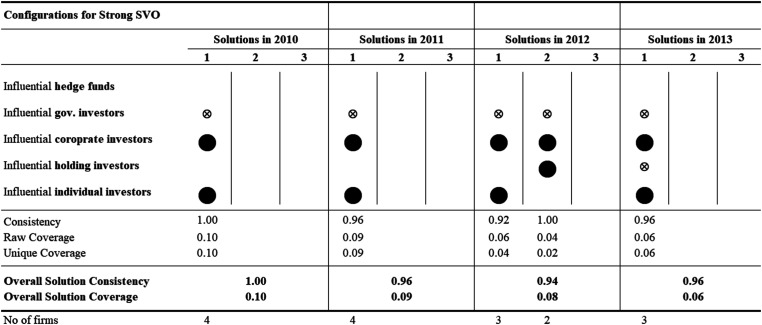
Configuration charts 2010–2013.

**Figure 4.**
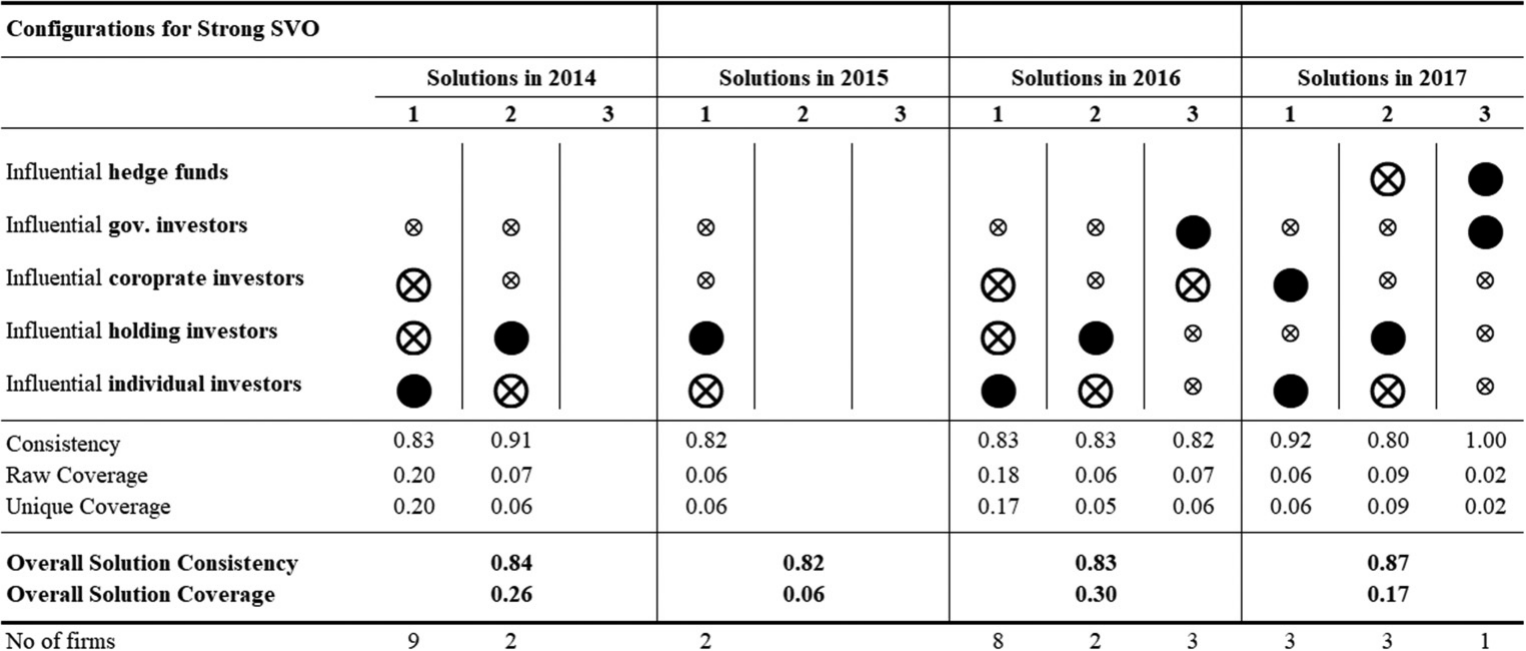
Configuration charts 2014–2017.

The results of the repeated cross-sectional QCA analyses—for each of the 12 years—identify several ownership configurations associated with strong SVO. Figures 2–4 show the configuration charts of the repeated cross-sectional QCA for each year between 2006 and 2017.^2^ Across the 12 years, we identify between one and four ownership configurations associated with strong SVO. The solutions show high levels of overall solution consistency (between 0.82 and 1.00) and low (0.3) to very low (0.06) overall solution coverage. Following standards of good practice in QCA, we ran the same repeated cross-sectional QCA for the negated outcome—that is, for weak SVO. Our results not only identify ownership configurations associated with strong (and weak) yearly SVO but also allow researchers to compare configurations over time and identify configurational themes, that is, recognizable temporal patterns of recurring combinations of explanatory conditions.

### Step 3: Defining Configurational Themes

In Step 3, the researcher prepares the results of the repeated cross-sectional QCA for use in the sequence analysis. This step requires the researcher to identify configurational themes by comparing the results of the various repeated cross-sectional QCA analyses. Across the years, a configuration may appear at every point in time and, hence, be considered recurring and temporally stable. Yet another configuration may experience changes in some of its elements, while other elements remain stable over time. In identifying configurational themes, the researcher must systematically account for changes in the co-occurrence of attributes over time.

To evaluate these changes, we propose a process of “configurational matching,” through which researchers systematically compare the results from different points in time to identify temporally stable configurations or parts of configurations. We propose two rules for configurational matching. First, some core and peripheral conditions may appear continuously throughout the observation period. Meanwhile, because the results of a panel data analysis may be biased to the specific time window that is analyzed, some core and peripheral conditions may only frequently appear and reappear for longer periods. In both instances, core and peripheral conditions that either appear continuously or frequently appear and reappear are temporally robust elements and can be considered “theme-defining” conditions. Second, other core conditions may appear recurrently within only one theme over a longer period. Because these conditions are temporally stable only in the context of a theme, they can be considered “theme-supporting” conditions.

Thus, through the process of configurational matching, researchers may empirically identify and define configurational themes. These configurational themes capture configurations of conditions that reoccur over time and researchers may distinguish between theme-defining from theme-supporting conditions. Yet, unlike the idea of an “orchestrating theme” that fully captures the integrative mechanism sufficient for an outcome at one point in time (e.g., Furnari et al., 2021; Miller, 1996), our concept of a “configurational theme” differs in that it is—at most points in time—not necessarily sufficient for an outcome to occur (unless the configurational theme happens to be identical to the orchestrating theme). Instead, a configurational theme requires every year additional conditions to become sufficient for an outcome.

**Figure 5.**
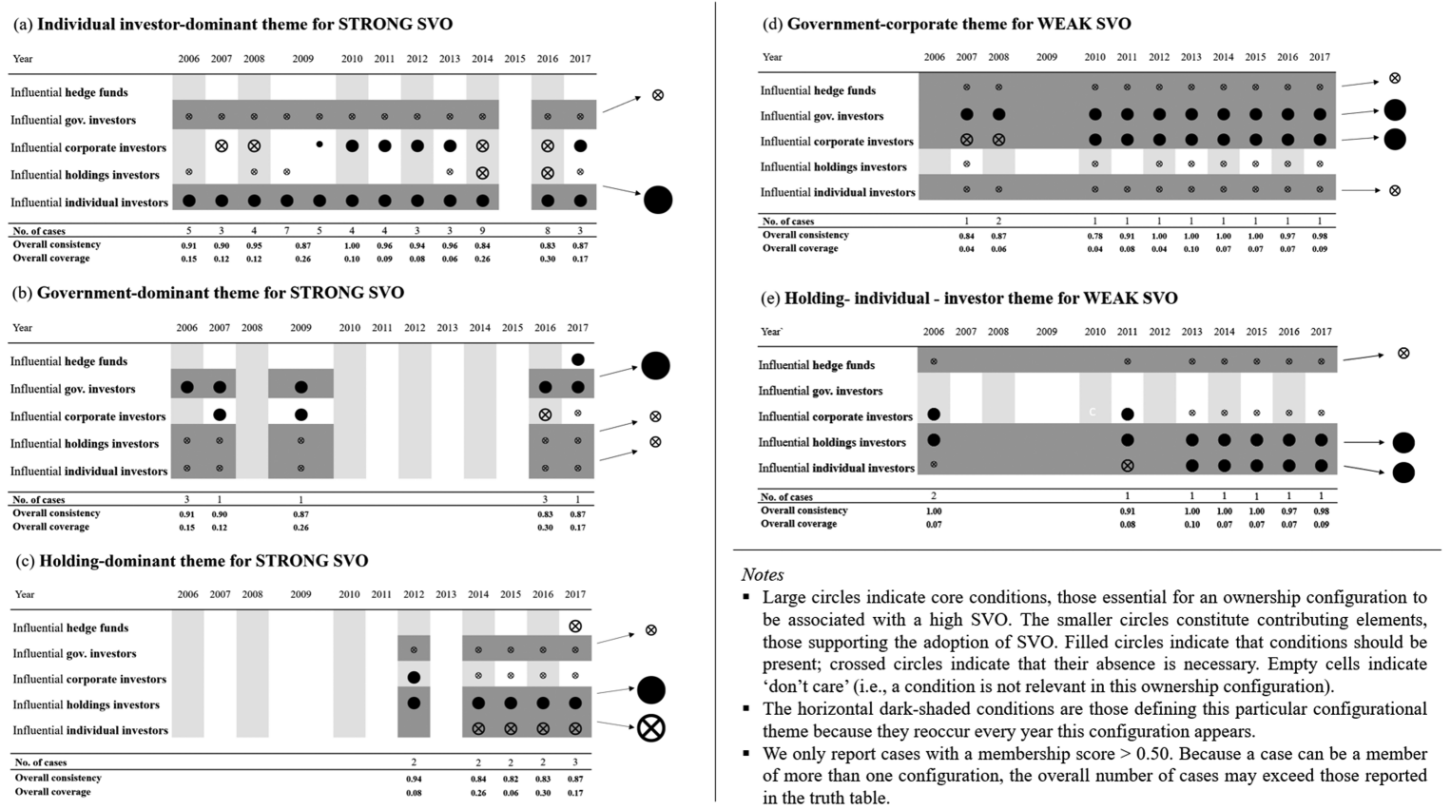
Configurational themes associated with a strong SVO (Panels (a)–(c) and with a weak SVO (Panels (d)–(e)).

*Note*. SVO = shareholder value orientation.

Panels (a)–(e) of Figure 5 show the results of the configurational matching procedure through which we systematically compared and grouped repeated cross-sectional QCA results. On the far-right edge of each panel, we depict the theme-defining and theme-supporting conditions constituting each theme. In total, we identified five configurational themes (i.e., combinations of core and peripheral conditions that are stable over time). Panels (a)–(c) show the themes associated with strong SVO. All three themes for strong SVO feature the presence of one dominant shareholder type (as a core condition) and the absence of one or two shareholder types (either as a core or a peripheral condition). Based on the dominant shareholder, we label the three themes “individual investor-dominant,” “government-dominant,” and “holding-dominant.”

Panels (d) and (e) visualize the themes associated with weak SVO. Both themes combine the presence of two dominant shareholders (as a core condition) with the absence of one or two shareholders (either as a core or a peripheral condition). For labeling themes for weak SVO, we also consider the presently dominating shareholders and identify one “government–corporate investors” and one “holding–individual investors” theme. Overall, Figure 5 depicts five themes that emerge as temporally stable configurations.

The results also indicate that, although all themes are structurally robust over time, they differ in their stability and regularity over time. Correspondingly, repeated cross-sectional QCA offers insights into the temporal dynamics of configurational themes. However, it does not reveal in what pathways companies adopt or diverge from some of these configurational themes. Before interpreting the results substantively, we explain how researchers integrate the results of the repeated cross-sectional QCA with sequence analysis.

### Step 4: Sequence Analysis

In Step 4, the researcher analyzes the sequences of when and how cases adopt configurational themes. The general protocol of sequence analysis comprises four steps: (1) generating a sequence dataset, (2) defining the indel and substitution costs from one sequence to another, (3) calculating the similarity (dissimilarity) between sequences, and (4) clustering sequences to identify groups of similar sequences. For our analysis, we conduct two separate sequence analyses, one for themes associated with a strong SVO and one for themes with weak SVO. This separation of analyses allows us to identify periods in which cases switch themes but also if they constantly have a strong (weak) SVO in their respective theme.

First, to create sequence data from the QCA, we use the panel data regarding the membership of each case in each theme as the starting point. Sequence data require defining the “states” of cases. These states must be stable over each time point in the observation period. We define states using the set membership scores of each company in each of the three configurational themes and in the outcome. We start by dichotomizing the membership scores for each configurational theme and use a membership score of above 0.5 to define a case that resembles this theme. Consequently, a company in our dataset may or may not resemble one of the three themes each year. Over the years, it may change its theme. For example, SAP belonged to the individual investor-dominant theme in 2006. During our observational period, the company's ownership structure was dominated by individual investors. The company is therefore a constant member of the individual investor-dominant theme from 2006 to 2017. In contrast, the ownership structure of Commerzbank changed in 2009 as a response to the acquisition of Dresdner Bank during the financial crisis and remained government dominant until the end of our observational period.

The calibrated data we use for the QCAs also provide information about a case's membership in the outcome (i.e., adopting a strong or weak SVO). This information allows us to distinguish cases that resemble a theme and show a strong SVO from those that resemble a theme but do not show a strong SVO. For those companies that do not resemble any of the three themes, we create a residual category. This procedure leads to a sequence dataset with eight states.^3^ Thus, the sequence dataset captures the company-level resemblance with each theme in each year and thereby contains all the necessary information required for the sequence analysis.

Second, one needs to specify the indel (i.e., insertion and deletion) and substitution costs. These costs indicate how “costly” the insertion or deletion is or how “costly” the entire substitution of an element in a sequence is. Researchers may define specific substitution costs by drawing on theoretical considerations and aspects related to particular attributes of a state in a data-driven fashion (e.g., using the frequency of transitions as a substitution cost; (Studer & Ritschard, 2016). For our example, we set the indel costs at 1 and the substitution costs at 2. These values indicate that the process of deleting one element and inserting another element is as costly as simply substituting an element. They also suggest that the same indel costs apply to all states and that sequences are comparable, even if they begin at different points in time.

Third, one calculates the dissimilarity between sequences. Sequence analysis assesses the dissimilarity between sequences by measuring how many insertions, deletions, or substitutions of elements are required to transform one sequence into another. To do so, one develops a dissimilarity matrix that measures and indicates the dissimilarity between every pair of sequences. For our illustration, we use optimal matching, the most common way to calculate the dissimilarity between sequences (Abbott & Tsay, 2000; Biemann & Datta, 2014; Brzinsky-Fay et al., 2006). Specifically, to develop the distance matrix, we use the Needleman–Wunsch algorithm (Abbott & DeViney, 1992; Biemann & Datta, 2014). In our study, with 54 companies in the dataset, the distance matrix spans 54 × 54 rows and columns (with zeros on the diagonal) and includes dissimilarity measures for all pairs of cases/sequences.

Fourth, one uses cluster analysis to analyze the dissimilarity matrix and the dissimilarity measures between all sequences and to identify those (generic) sequences that appear more frequently in the dataset. Cluster analysis in sequence analysis requires a clustering technique that clusters based on all the information included in the dissimilarity matrix. For our study, we use a cluster analysis with a Ward's linkage and stop the clustering after the identification of five clusters.^4^ Ward's linkage is a hierarchical clustering technique that explicitly builds on a dissimilarity matrix to form these groups. It forms clusters based on the closest or most similar observations, beginning with all observations in the data belonging to a different group. Its aim is to reduce the number of groups by grouping the two closest observations.

**Figure 6.**
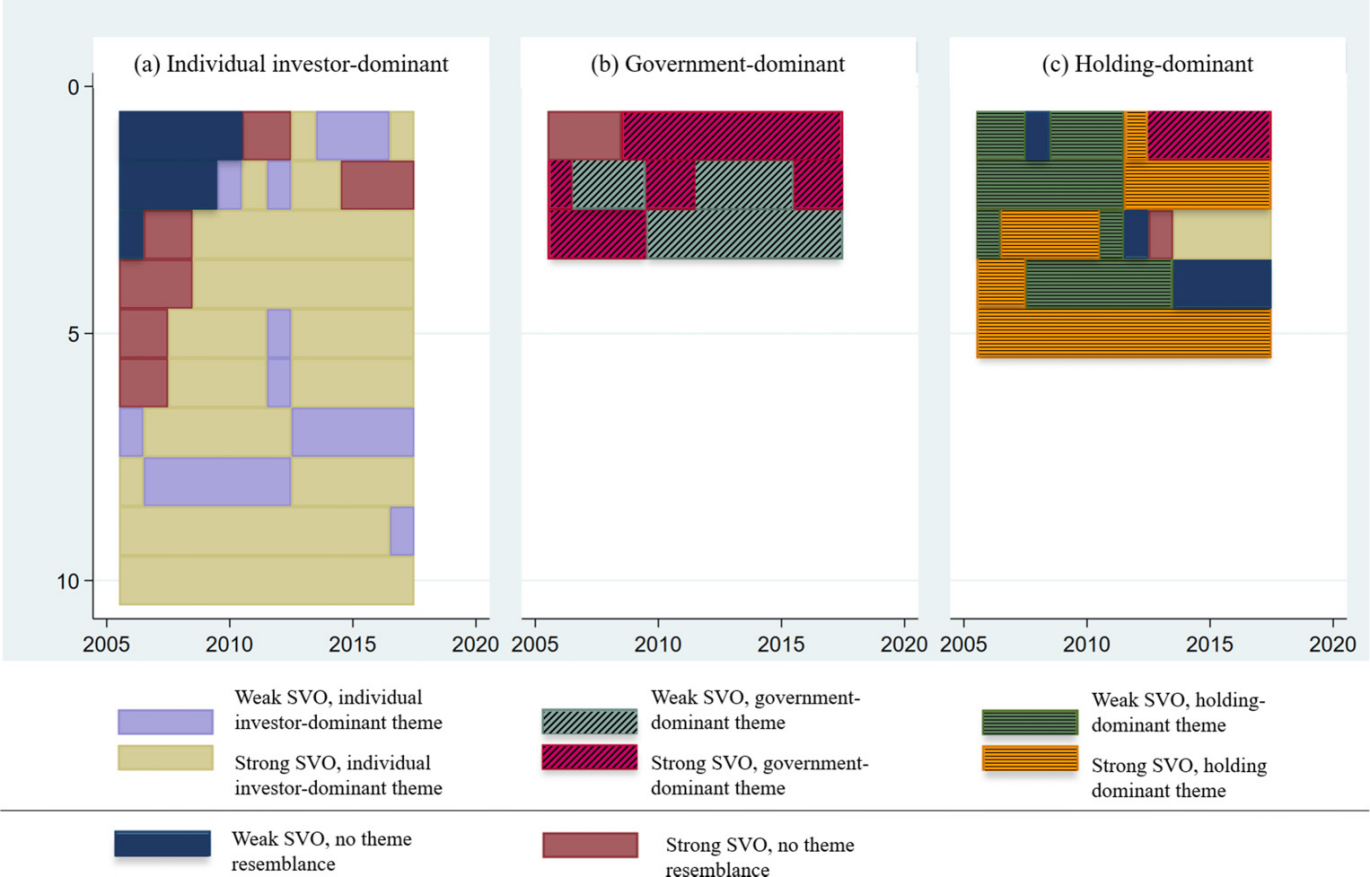
Clusters of sequences around the three configurational themes for a strong SVO.

*Note*. SVO = shareholder value orientation.

Figure 6 illuminates the results of the cluster analysis for our study on SVO.^5^ The horizontal bars in each panel correspond to a case, and the vertical axis indicates the years between 2006 and 2017. The colors in each bar represent the themes to which the case belongs in each year.^6^ Panels (a)–(c) in Figure 6 are dominated by firms that have adopted one configurational theme associated with a strong SVO. Yet, as the color bars indicate, firms adopting other themes and exhibiting a weak SVO also appear in these three panels. Panel (a) contains companies that resemble the individual investor-dominant theme. As the panel shows, companies appear to either switch from the residual category to the investor-dominant theme or have adopted this before our observation period. Panel (b) is the smallest of the three and reveals companies associated with the government-dominant theme. Only one company switches to this theme during our observation period, and phases of weak SVO appear frequently in this cluster.

Finally, Panel (c) has companies that have adopted the holding-dominant theme. We observe that while some companies in this cluster adopted the theme from the beginning of the observation period, toward its end, several firms opted out of the holding-dominant theme. In summary, the sequence analysis identifies three clusters of sequences that closely correspond to the configurational themes identified in Step 3. These clusters not only differ in how the number of firms pursuing these pathways adopt strong SVO but also display variations in how firms switch within and between themes.

### Step 5: Interpreting C^2^PA Results

In Step 5, one uses the collective findings from both the QCA and the sequence analysis to address the research inquiries. In our illustration, we posed several questions pertaining to the temporal dynamics among the ownership configurations associated with an SVO. We inquired whether diverse ownership configurations are associated with the adoption of SVO, whether companies maintain a strong SVO amid changes in their ownership structures, how these configurations emerge and diffuse over time, and whether they undergo internal and external temporal fluctuations.

#### The Individual-Investor-Dominant Theme

The first configurational theme associated with a strong SVO is characterized by the dominance of individual investors as a foundational element, with a notable absence of government investors. Both conditions are theme-defining as they appear almost continuously as temporally highly stable parts of the theme. Figure 6(a) shows that over successive periods, this theme exhibits occasional appearances of other shareholder types, such as holding investors, albeit sporadically, or variations in the significance of corporate investors. However, these alternate shareholder types lack temporal stability and are not integral components of this first theme. We thus do not identify any theme-supporting conditions for this theme. The results of the sequence analysis, revealed in Figure 6(a), also demonstrate that six out of 10 firms display no thematic resemblance initially but gradually adopt the investor-dominant theme over time. Subsequently, a pattern emerges wherein firms sustain the investor-dominant theme across extended periods. Thus, the individual-investor-dominant theme appears particularly attractive and coherent for firms. Once they adhere to this investor-dominant theme, they merely oscillate between embodying a strong or weak SVO.

Several firms in our dataset exemplify the individual-investor-dominant theme, including BMW, MLP, and Continental. In these cases, a single dominant individual investor holds sway over the ownership structure. For instance, the Quant-Klatten family controls 45% of BMW, the Lautenschlaeger family holds 54% of MLP, and the Schaeffler family owns 46% of continental. Notably, our findings indicate that the individual-investor-dominant theme does not encompass several dominant investors, but a single one; the remaining shares are fragmented among numerous small free-floating shares.

Overall, corroborating research on SVO, our results indicate that the individual-investor-dominant theme is the most frequent theme and most closely resembles the ownership structure most widely discussed in the SVO literature. At the same time, the integration of QCA with sequence analysis offers important new insights. First, the configurational theme is highly robust over time. The temporal stability of the individual-investor-dominant theme requires balancing not only the presence but notably also the absence of a particular shareholder type. Second, this configuration of shareholders appears particularly attractive, as firms show a clear tendency to adopt (or move toward) the individual-investor-dominant theme. Third, contrary to research on shareholder typologies, hedge funds—typically viewed as influential proponents of SVO—appear to play an inconspicuous role in this context.

#### The Government-Dominant Theme

Characteristic of the second configurational theme associated with a strong SVO is dominant governmental investors, alongside the absence of two other shareholders, namely, holdings and individual investors. We identify all three conditions as theme-defining because they frequently appear and reappear as temporally robust elements. As depicted in Figure 6(b), within this government-dominant theme, the presence, absence, or relevance of all other shareholder types (such as investors or holdings) may vary across years. Over time, this theme calls for a delicate balance between present and absent shareholder types, with one shareholder exerting influence over the others. Yet, unlike the individual-investor-dominant theme, this configuration comprises three structurally complex components.

The results from the sequence analysis also indicate that the government-dominant theme appears less frequently than the other themes. We observe a relatively stable occurrence until 2009. However, between 2010 and 2015, both shifts in shareholder structures or alignment with the adoption of SVO result in the disappearance of this theme. From 2016 onward, the government-dominant theme reemerges. The sequence analysis results depicted in Figure 6(b) reveal that while one company consistently exhibits a strong SVO, the other two fluctuate between weak and strong orientations. Thus, the sequence analysis complements the cross-sectoral QCA and aids in explaining temporal variations in configurational themes.

One case that closely resembles the government-dominant theme is Deutsche Telekom, Europe's largest telecommunications company, which emerged in 1995 as the successor to Germany's state-owned postal company. During the telecommunications revolution of the 1990s, Deutsche Telekom achieved considerable success both domestically and internationally. Despite its state ownership, the company progressively embraced a strong market orientation. The privatization process initiated in 2005 bolstered its ongoing market-focused approach. However, throughout the 2010s, the company faced tumultuous periods marked by various splits and mergers, maintaining the continued dominance of state-owned entities. (For example, in 2022, the federal government held 13.8% ownership, and Kreditanstalt für Wiederaufbau (KFW Development Bank), a German state-owned investment bank, held 16.6%, while increasing the proportion of free-floating shares in the market to nearly 60%.)

The case of Deutsche Telekom shows less frequent occurrence and greater temporal variability of the government-dominant theme. Despite its variability, it emerges as a configurational theme relevant to firms with a legacy. The presence of government investors, contrary to the prevailing literature on SVO and shareholder orientations, highlights the government-investor-dominant theme, resting on a deterrence balance wherein one dominant shareholder influences the extent to which the company adopts an SVO. The fact that this theme demonstrates longer periods of decline (e.g., between 2010 and 2015) and reemergence (i.e., in 2016) also suggests that it may be more strongly influenced by exogenous factors than the individual-investor-dominant theme.

#### The Holding-Dominant Theme

The third configurational theme linked with a strong SVO is marked by the presence of holding investors, the absence of individual investors (two core conditions), and the absence of governmental investors (a peripheral condition). Whereas we identify the presence of holding investors and the absence of governmental investors as theme-defining conditions (they continuously appear over the years), we identify the absence of individual investors as a theme-supporting condition as it reoccurs only within the context of the theme. Given this specific ownership structure, we term this pattern the “holding-dominant theme.” Figure 6(c) shows that the holding-dominant theme occurs less frequently than the individual-investor-dominant one. Similar to the preceding two themes associated with a strong SVO, there are notable conflicting dynamics between the presence of one type of investor and the absence of several others. Pivotal to the enduring stability of the holding-dominant theme could be an exclusive balance between a holding investor and all other types of shareholders.

The cross-sectional QCA analyses and the sequence analysis offer further insights into the stability of this third theme over time. Figure 6(c) illustrates that the holding-dominant theme only surfaces in 2012 during the latter part of our observation span. Hence, akin to the government-dominant theme, it seems prone to temporal irregularities. However, instead of intermittent occurrences (as seen in the government-dominant theme), we witness the emergence of a new ownership structure associated with a strong SVO. Furthermore, the sequence analysis indicates similar patterns of change as observed for the government-dominant theme. Figure 6(c) reveals that some companies maintain their ownership structure but oscillate between strong and weak SVO. Unlike firms characterized by a government-dominant theme, those aligned with the holding theme undergo alterations in their ownership structures and in their SVO.

A closer examination of some typical cases representing the holding-dominant theme illuminates the dynamics behind why this theme is associated with a strong SVO. Two illustrative cases are Beiersdorf (a multinational manufacturer of personal care products, including brands like Nivea), owned 51% by the holding Maxinvest, and Hannover Rück (one of the largest reinsurance companies globally), owned 51% by Talanx AG and approximately 38% by other institutional investors. In particular, the case of Beiersdorf highlights the inherent dynamics of the holding-dominant theme. Since the early 2000s, Beiersdorf has been subject to significant hostile takeover attempts (e.g., by Procter & Gamble). These endeavors spark intense debates and discussions about the company's mission. Despite minor shifts in its ownership structure, the dominance of one holding owner persists over the years. However, its clear shareholder focus diminishes as the primary holding company that owns Beiersdorf seeks to preserve their role in benefiting local communities through tax contributions and quality employment opportunities.

Overall, the holding-dominant theme reveals a temporally stable ownership structure associated with a strong SVO held together primarily by a holding company. Our case evidence emphasizes the additional endorsement of other institutional investors. Notably, this ownership structure entails opposing tensions that arise between individual investors and governmental entities. Our results also provide evidence of the emergence of new configurational themes. Unlike the government-dominant paradigm, shifts in temporal dynamics regarding the adoption of a strong SVO appear more contingent upon changes in firm ownership structures and more reflective of owners’ evolving interests.

#### Two Configurational Themes Associated with Weak SVO

We also conducted a separate C^2^PA to identify and explain the temporal dynamics among the configurational themes associated with a weak SVO. In Figure 6(d)–(e), we identify two configurational themes with weak SVO. Yet, due to the limited number of cases, the sequence analysis within the C^2^PA fails to discern specific patterns linking changes in ownership structure to shifts in firms’ SVO.

The first configurational theme associated with a weak SVO is characterized by the presence of governmental investors and corporate investors as core conditions, alongside the absence of hedge funds and individual investors as peripheral conditions. As depicted in Figure 6(d), this government-corporate theme persists throughout our observation period, albeit infrequently. All conditions appear as theme-defining conditions (they continuously appear over the years) but shift between absent and present influential corporate investors between 2008 and 2010 suggests a more fundamental shift in this configurational theme. Moreover, unlike themes linked with a strong SVO, this theme exhibits greater structural complexity. Rather than balancing the interests of one dominant shareholder type, it necessitates balancing the interests of two dominant shareholders: governmental and corporate investors.

One corporation that closely aligns with the government-corporate-investor theme is Salzgitter, one of Europe's largest steel producers. Historically, during the 1960s, the company was also the largest state-owned corporation globally. However, since 1990, significant portions of Salzgitter have undergone privatization. Today, Salzgitter is co-owned by the local state (approximately 26%), its employees (around 10%), a local institutional investor (25%), and other corporate investors (21%). Notably, all major owners are deeply rooted in the local community. Consequently, Salzgitter actively supports the local economy and communities, with its focus appearing to be less shareholder oriented, at least in the short term. Clearly, Salzgitter's history and local embedding explain how this ownership structure leads firms to suppress a strong focus on shareholders in favor of a broader orientation toward various stakeholders.

The second ownership theme linked with a low SVO is defined by the presence of holdings and individual investors as core conditions, alongside the absence of hedge funds as a peripheral condition. Similar to the government-corporate theme, this holding-individual-investor theme arises sporadically and displays temporal irregularities, as depicted in Figure 6(e), notably emerging post-2012. The case that closely resembles this theme is MLP, a company that represents the individual-investor-dominant theme associated with a strong SVO.^7^ The presence of MLPs in both themes can be traced to years of shareholder tensions. In 2009, Swiss Life, a Swiss insurance company, acquired significant stakes in MLP against the founders’ wishes. This period witnessed a marked decline in MLP's profits due to unfavorable financial market conditions. Subsequently, through important share transactions, MLP replaced Swiss Life with Talanx, a major financial holdings company, altering not only its ownership structure but also its SVO.

In summary, our analysis uncovers two configurational themes associated with weak SVO. Through cross-sectional QCA, we not only unveil their configurational structure but also shed light on the temporal dynamics shaping them. Nevertheless, our findings yield intriguing insights. The structural divergence of configurational themes for weak SVO is apparent, as they involve balancing the interests of two complementary shareholder types. In contrast, the themes for strong SVO are all defined by a dominant shareholder type that ensures the exclusion of others. Additionally, our case-based evidence indicates that companies with weak SVO often have strong local ties, prompting them to prioritize a wider range of stakeholders, including local governments, for aspects such as tax revenue and employment concerns.

## Discussion

In this paper, we introduce a new technique, termed C^2^PA, designed to analyze configurational patterns over time. C^2^PA blends QCA with sequence analysis, facilitating the disentanglement of temporal dynamics at both the theme and case levels. To demonstrate C^2^PA, we examined the dynamics surrounding the adoption of strong and weak SVO across 54 companies listed on the German HDAX from 2006 to 2017. By showcasing C^2^PA's application, we highlight its advantages in analyzing temporal dynamics that are often challenging for traditional QCA methods.

### Contributions to the Methods on Time and QCA

C^2^PA directly constitutes an important contribution to the methodological literature on time and QCA. As a novel five-step sequential mixed-methods design that uses the results of the QCA as input for sequence analysis, it offers unique opportunities to study temporal dynamics from a configurational perspective, revealing patterns of stability, emergence, and decline of configurational phenomena. Alongside C^2^PA, we introduce the notion of a “configurational theme” as a recognizable temporal pattern of recurring combinations of explanatory conditions. The concept of a configurational theme closely relates to and possibly extends the idea of an “orchestrating theme.” A configurational theme adopts a temporal lens and decomposes orchestrating themes of configurations into essentially temporally stable parts and supporting parts with weaker temporal stability. Thus, the concept of a configurational theme should advance configurational theorizing from a temporal perspective and in so doing offer new opportunities for capturing the evolving temporal dynamics among configurational phenomena.

We also outline the associated approach of configurational matching for the identification of configurational themes. Configurational matching distinguishes the temporally stable parts of configurations from unstable parts. More specifically, we distinguish theme-defining conditions (i.e., temporally highly stable parts of configurations) from theme-supporting conditions (i.e., substantively relevant, and recurring parts of configurations within a theme), aiding in the empirical identification of configurational themes. Our proposal for configurational matching is probably only one possible approach, and we anticipate that researchers will improve and adjust our configurational matching rules to their research questions and data structures. Nonetheless, proposing configurational matching is important because it outlines a first formalized step to identify similarities and differences among organizational configurations over time and offers researchers new ways to decompose configurations by their temporal stability.

Overall, C^2^PA offers insights that surpass those achievable through either QCA or sequence analysis alone. While a repeated cross-sectional QCA would produce identical outcomes as C^2^PA if cases remained stable over time, the scenario changes when configurations evolve dynamically. In such cases, revealing configurational themes becomes crucial for explaining temporal dynamics and identifying pivotal moments in which cases appear influenced by exogenous effects. Moreover, integration with sequence analysis becomes essential because it enables researchers to discern if firms switch within or between themes, identify pivotal switching instances, and thereby explain adoption patterns at the case level. In essence, C^2^PA stands as the sole approach capable of systematically unveiling temporal patterns at both the theme and case levels, facilitating systematic clustering.

### Contributions to Research on SVO

Our illustrative study of SVO reveals the unique explanatory power of C^2^PA. The results of our C^2^PA corroborate research on SVO and identify one highly robust and prevalent ownership configuration clearly associated with the strong adoption of SVO. The dominance of individual investors emerges as a structurally straightforward and temporally robust theme that closely aligns with the ownership structure conventionally linked to SVO. Simultaneously, our results make several contributions to research on SVO. First, they unveil not just one but multiple ownership structures associated with SVO. Specifically, we identified three themes associated with strong SVO and two with weak SVO. This insight underscores the concept of equifinality among ownership structures not only in a static context but also across temporal dimensions, hinting at potentially overlooked alternative configurations in prior SVO research.

Second, the structural differences between configurational themes linked to strong and weak SVO are striking. All themes associated with strong SVO showcase a concentrated ownership model, wherein a single shareholder type dominates the theme to such an extent that other types are consistently deterred from assuming ownership roles. Conversely, themes associated with weak SVO exhibit a distinct balanced ownership configuration characterized by the involvement of at least two shareholder types sharing ownership responsibilities. This observation not only emphasizes the role of causal asymmetry among configurational themes between strong and weak SVO but also raises important substantive questions. Considering the increasing scrutiny of SVO, if one conceptualizes configurational themes linked to weak SVO as governance forms oriented toward “stakeholder” value, our findings underscore the necessity for further research to develop insights into more inclusive shareholder structures. Such structures could balance the complementary perspectives of multiple shareholder types, thereby addressing criticisms and fostering a more holistic approach to governance.

Third, C^2^PA unveils significant temporal dynamics at both the theme and case levels. We demonstrate that the most prevalent ownership configuration, long under the scrutiny of SVO research, exhibits remarkable temporal stability. Conversely, other ownership configurations associated with both strong and weak SVO display varied temporal patterns characterized by stability, emergence, and decline at different junctures. Through C^2^PA, we not only pinpoint representative cases but also highlight pivotal years when external influences shaped company trajectories. Importantly, our results emphasize the role of a company's legacy and its integration within a local economy as critical contingency factors in theories elucidating SVO adoption. In essence, we illustrate how C^2^PA provides organizational researchers with unique opportunities to analyze configurational phenomena over time.

### Applicability, Limitations, and Future Research Opportunities

We illustrated C^2^PA with a relatively simple example. However, interested researchers should consider several factors when using C^2^PA. First, C^2^PA proves particularly valuable for investigating the temporal dynamics of organizational configurations or the configurational structure of parallel processes. These research objectives require specific types of data. For example, in our illustration, we used a balanced panel of 54 companies, revealing some stable ownership configurations and sequence patterns over time. The simplicity of such a balanced panel was instrumental for highlighting C^2^PA's applications and opportunities. Nonetheless, applying C^2^PA with an unbalanced panel should be unproblematic given a substantial overlap in the case population across waves. Yet, researchers should treat the analysis of repeated cross-sectional data (e.g., pseudo panels aggregating individual data to groups that a researcher can follow over time) with caution because this requires a match between the configurational theme and the aggregate data (as opposed to individual observation), thereby leading to insufficient data for the sequence analysis. Thus, while researchers may use C^2^PA to analyze smaller and unbalanced panel datasets, depending on the quality and structure of the dataset, they may face methodological challenges that this paper has not foreseen.

Furthermore, C^2^PA provides researchers with opportunities to align their theoretical frameworks closely with modeling decisions throughout the stages of the research process. For example, in QCA, calibration may significantly influence the occurrence of configurations, thereby affecting the definition of configurational themes (i.e., the states in the sequence analysis). Hence, researchers must carefully assess the temporal sensitivity of individual conditions, such as anchoring on legal requirements and choosing between time-insensitive and time-sensitive calibration methods. It is crucial for research settings involving temporally bounded configurational phenomena or conditions subject to temporal variations, such as essentially contested concepts (e.g., Meyer & Höllerer, 2010; Okoye, 2009), to ensure that calibration approaches do not compromise the requirements of sequence analysis. In sequence analysis, one could also leverage different information from QCA to define indel and substitution costs. This could involve tying indel and substitution costs to structural differences between themes, for example, by asking how many conditions must switch to make two themes identical. Clearly, C^2^PA offers several intriguing avenues for more closely aligning a researcher's theorizing with modeling choices.

As one of the first steps toward innovative techniques at the intersection of process and configurational research, C^2^PA highlights unexplored opportunities to integrate sequence analysis and QCA. Despite process methods primarily aiming to analyze temporal dynamics and change sequences, they share numerous conceptual principles and tools with configurational research. Consequently, there are promising prospects for leveraging these similarities in future research endeavors to cultivate supplementary methods.

First, C^2^PA adopts a mixed-methods approach by integrating QCA with sequence analysis within a structured five-step model. The effectiveness of mixed-methods designs hinges on the degree and timing of method integration within the research process (Tashakkori & Teddlie, 2008). Portrayed in our example is the intricate conceptual relationship between calibration in QCA and the delineation of states in sequence analysis, which presents opportunities for deeper integration. For example, insights from QCA results regarding the unique and raw coverage of configurations can be interpreted as indicators of configurational theme diffusion. A substantial intersection of coverage between two configurations could signify high similarity, with the shared coverage measure serving as a proxy for substitution costs. Researchers may thus consider using data on set intersections to develop new and more integrated measures for indel and substitution costs (Ritschard & Studer, 2018).

Second, C^2^PA offers additional opportunities for case selection. In QCA, researchers have proposed various strategies such as identifying typical or deviant cases based on QCA analysis outcomes (Ragin, 2008; Schneider & Rohlfing, 2016; Schneider & Wagemann, 2013). Similarly, in sequence analysis, scholars advocate purposeful case selection techniques informed by sequence analysis results (Borgna & Struffolino, 2018; Le Roux et al., 2020). We argue that C^2^PA enhances these opportunities by pinpointing theoretically significant cases according to their alignment with a configurational theme and outcome, as well as their centrality within sequence clusters. Such a selection method holds promise for in-depth case study approaches, such as process tracing, as it integrates temporal dynamics—central to process tracing (Beach & Rohlfing, 2018)—into the case selection criteria.

Third, we introduced a novel analytical technique for examining the temporal dynamics of configurational phenomena. However, the efficacy of empirical studies relies heavily on the alignment between theories and methods (Delbridge & Fiss, 2013). Currently, although researchers in each domain occasionally borrow conceptual insights from the other (Abbott & DeViney, 1992; Fiss & Zajac, 2004; Misangyi et al., 2017; Raisch et al., 2018), few theories integrate both process and configurational perspectives. For example, organizational researchers have developed theories on various processes and their salient features (Monge, 1990; Van de Ven & Poole, 1995).

Yet, questions remain regarding whether patterns of change in nonconfigurational phenomena mirror those in configurational phenomena. How do concepts such as structural equifinality in configurations compare to process equifinality, and how do the central features of processes relate to core and peripheral conditions in configurational research? This suggests substantial opportunities for developing integrative theories of the processes of configurational change or the configurations of processes. Future research engaging in more theoretical exploration and combining conceptual and analytical considerations could uncover further applications for C^2^PA by jointly studying organizational processes and configurations.

Finally, we argue that C^2^PA offers ample opportunities to make substantive contributions to management research. In exploring organizational phenomena, scholars across management research domains have increasingly embraced a configurational lens. Our study, in the context of SVO research, has illustrated the potential contributions of C^2^PA to governance research. Yet, scholars in other fields of management research may also be interested in asking similar questions about the temporal dynamics of configurational phenomena. For example, research on organizational forms, business models, and ecosystems has frequently conceptualized organizational phenomena as configurational constructs, employing typological theorizing to delineate generic structures (Miles & Snow, 1984; Murray, 1988; Short et al., 2008; Zajac & Shortell, 1989). Nevertheless, the effectiveness of these generic shapes may hinge on broader macroeconomic shifts, such as economic downturns or trade disputes. Through C^2^PA, scholars can probe the emergence and structural stability of configurations over time while assessing the strength of the theme–performance nexus.

Moreover, research on corporate governance and HRM systems has traditionally viewed organizational practices as bundles necessitating internal alignment for optimal performance (Aguilera et al., 2015; MacDuffie, 1995; Meuer, 2017). However, the efficacy of these bundles may vary based on internal and external contingencies, suggesting that ideal configurations may evolve over time. Similarly, in research on innovation studies, the concept of a “policy mix” has gained prominence, referring to a collection of policy tools and interventions (Flanagan et al., 2011; Rogge & Reichardt, 2016). This field contends that policy instruments vary in complexity and effectiveness over time, emphasizing the significance of “policy windows” that provide unique intervention opportunities (Flanagan et al., 2011). C^2^PA would enable researchers to investigate temporal stability and opportunities within policy mixes.

Last, C^2^PA may also hold promise for refining and broadening scholarly insights into organizational processes within management research. The prevailing process analytical methods have predominantly focused on elucidating directly observable phenomena, such as career advancements or policy implementations (e.g., Abbott & DeViney, 1992; Biemann & Datta, 2014; Pentland et al., 2020). Yet, C^2^PA introduces a novel technique for probing process-oriented inquiries regarding configurational, latent, and less observable constructs (Aguinis & Molina-Azorín, 2015; Meuer & Rupietta, 2017a). Researchers may extend the systematic approach outlined in process tracing to analyze multiple case studies, moving beyond singular case examinations. Interestingly, some process scholars have advocated for conceptualizing processes as “multiple parallel progressions” (Van de Ven, 2007, p. 193) aligning with configurational thinking. C^2^PA may offer a structured way to comparatively study multiple processes and extend the notion of configuration equifinality into a temporal framework.

### Conclusion

With the growing interest in management research studying organizational phenomena from a configurational perspective, researchers increasingly ask questions concerning the temporal dynamics of organizational phenomena. Thus far, however, QCA, as the dominant approach for studying configurational phenomena, has not allowed researchers to empirically study the extent to which configurational phenomena are temporally stable, how they emerge or decline, and whether internal or external forces alter their shape over time. In this paper, we present C^2^PA—a technique that integrates QCA with sequence analysis—as an approach that allows researchers to analyze such questions empirically. We introduce the idea of a “configurational theme,” a recognizable temporal pattern of recurring combinations of conditions, and an associated procedure of configurational matching for defining themes. The configurational theme allows us to link QCA and sequence analysis to analyze temporal patterns in configurations. We provide both a protocol and an example that guides researchers using C^2^PA. With C^2^PA, we offer a new mixed-methods technique that enables the integrated analysis of complex temporal and configurational patterns.

## Supplemental Material

sj-docx-1-orm-10.1177_10944281241259075 - Supplemental material for Comparative Configurational Process Analysis: A New Set-Theoretic Technique for Longitudinal Case Analysis Supplemental material, sj-docx-1-orm-10.1177_10944281241259075 for Comparative Configurational Process Analysis: A New Set-Theoretic Technique for Longitudinal Case Analysis by Christian Rupietta and Johannes Meuer in Organizational Research Methods

sj-docx-2-orm-10.1177_10944281241259075 - Supplemental material for Comparative Configurational Process Analysis: A New Set-Theoretic Technique for Longitudinal Case Analysis Supplemental material, sj-docx-2-orm-10.1177_10944281241259075 for Comparative Configurational Process Analysis: A New Set-Theoretic Technique for Longitudinal Case Analysis by Christian Rupietta and Johannes Meuer in Organizational Research Methods

sj-docx-3-orm-10.1177_10944281241259075 - Supplemental material for Comparative Configurational Process Analysis: A New Set-Theoretic Technique for Longitudinal Case Analysis Supplemental material, sj-docx-3-orm-10.1177_10944281241259075 for Comparative Configurational Process Analysis: A New Set-Theoretic Technique for Longitudinal Case Analysis by Christian Rupietta and Johannes Meuer in Organizational Research Methods

## Notes

1 Raw consistency indicates the proportion of cases in a truth table row that displays the outcome. PRI consistency guards against reporting false positive configurations by additionally considering the degree to which a configuration is not simultaneously sufficient for the occurrence and non-occurrence of the outcome.

2 In line with standards of good practice, we calculated the repeated-cross-sectional QCA for the absence of strong SVO. We applied the same raw consistency, PRI, and frequency cutoffs (only in 2010 we reduced the raw consistency cutoff to 0.75). We calculate the intermediate solution using the absence of strong ownership of hedge funds and the presence of strong government ownership as directional expectations. These results are available in Supplementary file C. The yearly truth table are available from the authors upon request.

3 The number of states that emerge from configurational themes is calculated as follows: (CT+1)*2, where CT corresponds to the number of configurational themes.

4 We follow Biemann and Datta (2014) specification of the cluster analysis. We used the Calinski-Harabasz pseudo-F as a stopping rule for the clustering. The results suggested one stop the clustering at three clusters. To improve the interpretability of the clusters, we stop the clustering at five clusters. Clusters 2, 3, and 4 would have been integrated to form one cluster if we had followed the recommendation of the stopping rule.

5 We also conducted the sequence analysis for a weak SVO. However, because only very few firms consistently exhibit any of the two configurational themes for a weak SVO, the analysis provides only limited additional insights. The results are available on request.

6 We excluded two of the five clusters we identified with cluster analysis because they mainly show results for companies with either strong or weak SVO in the residual category.

7 C^2^PA can identify situations of temporal causal asymmetry. In this situation, a case can be member of a configuration associated with the presence of the outcome, while in a different year, the same case can be a member of a configuration associated with the absence of the outcome.

## References

[bibr1-10944281241259075] Abbott A. (1990). A primer on sequence methods. Organization Science, 1(4), 375–392. 10.1287/orsc.1.4.375

[bibr2-10944281241259075] Abbott A. DeViney S. (1992). The welfare state as transnational event: Evidence from sequences of policy adoption. Social Science History, 16(2), 245–274. 10.2307/1171289

[bibr3-10944281241259075] Abbott A. Tsay A. (2000). Sequence analysis and optimal matching methods in sociology: Review and prospect. Sociological Methods & Research, 29(1), 3–33. 10.1177/0049124100029001001

[bibr4-10944281241259075] Aguilera R. V. Desender K. Bednar M. K. Lee J. H. (2015). Connecting the dots: Bringing external corporate governance into the corporate governance puzzle. Academy of Management Annals, 9(1), 483–573. 10.5465/19416520.2015.1024503

[bibr5-10944281241259075] Aguinis H. Molina-Azorín J. F. (2015). Using multilevel modeling and mixed methods to make theoretical progress in microfoundations for strategy research. Strategic Organization, 13(4), 353–364. 10.1177/1476127015594622

[bibr6-10944281241259075] Aisenbrey S. Fasang A. E. (2010). New life for old ideas: The “second wave” of sequence analysis bringing the “course” back into the life course. Sociological Methods & Research, 38(3), 420–462. 10.1177/0049124109357532

[bibr7-10944281241259075] Aktiengesetz (2023). German Stock Corporation Act.

[bibr8-10944281241259075] Andresen M. Biemann T. Pattie M. W. (2015). What makes them move abroad? Reviewing and exploring differences between self-initiated and assigned expatriation. International Journal of Human Resource Management, 26(7), 932–947. 10.1080/09585192.2012.669780

[bibr9-10944281241259075] Arellano M. C. Meuer J. Netland T. H. (2021). Commitment follows beliefs: A configurational perspective on operations managers’ commitment to practice adoption. Journal of Operations Management, 67(4), 450–475. 10.1002/joom.1130

[bibr10-10944281241259075] Aversa P. Furnari S. Haefliger S. (2015). Business model configurations and performance: A qualitative comparative analysis in Formula One racing, 2005–2013. Industrial and Corporate Change, 24(3), 655–676. 10.1093/icc/dtv012

[bibr11-10944281241259075] Baumgartner M. (2013). Detecting causal chains in small-n data. Field Methods, 25(1), 3–24. 10.1177/1525822X12462527

[bibr12-10944281241259075] Baumgartner M. Epple R. (2014). A coincidence analysis of a causal chain: The Swiss minaret vote. Sociological Methods & Research, 43(2), 280–312. 10.1177/0049124113502948

[bibr13-10944281241259075] Beach D. Rohlfing I. (2018). Integrating cross-case analyses and process tracing in set-theoretic research: Strategies and parameters of debate. Sociological Methods & Research, 47(1), 3–36. 10.1177/0049124115613780

[bibr14-10944281241259075] Bell R. G. Filatotchev I. Aguilera R. V. (2014). Corporate governance and investors’ perceptions of foreign IPO value: An institutional perspective. Academy of Management Journal, 57(1), 301–320. 10.5465/amj.2011.0146

[bibr15-10944281241259075] Biemann T. Datta D. K. (2014). Analyzing sequence data: Optimal matching in management research. Organizational Research Methods, 17(1), 51–76. 10.1177/1094428113499408

[bibr16-10944281241259075] Blair M. M. (2003). Shareholder value, corporate governance, and corporate performance. In: Cornelius P. Kogut B. (Eds.), Corporate governance and capital flows in a global economy (pp. 53–82). Oxford University Press.

[bibr17-10944281241259075] Borgna C. Struffolino E. (2018). Unpacking configurational dynamics: Sequence analysis and qualitative comparative analysis as a mixed-Method Design. In Gilberg Ritschard M. S. (Ed.), Sequence analysis and related approaches: Innovative methods and applications (pp. 167–184).

[bibr18-10944281241259075] Brzinsky-Fay C. Kohler U. (2010). New developments in sequence analysis. Sage Publications.

[bibr19-10944281241259075] Brzinsky-Fay C. Kohler U. Luniak M. (2006). Sequence analysis with Stata. The Stata Journal, 6(4), 435–460. 10.1177/1536867X0600600401

[bibr20-10944281241259075] Carberry E. J. Zajac E. J. (2021). Unpacking the dynamics of a contested practice: The case of executive compensation and the shareholder value orientation in the USA. Socio-Economic Review, 19(1), 157–187. 10.1093/ser/mwaa026

[bibr21-10944281241259075] Caren N. Panofsky A. (2005). TQCA: A technique for adding temporality to qualitative comparative analysis. Sociological Methods & Research, 34(2), 147–172. 10.1177/0049124105277197

[bibr22-10944281241259075] Delbridge R. Fiss P. (2013). Editors comments: Styles of theorizing and the social organization of knowledge. Academy of Management Review, 38(1), 325–331. 10.5465/amr.2013.0085

[bibr23-10944281241259075] Denning S. (2017, July 17). Making sense of shareholder value: ‘The world's dumbest idea’. *Forbes Magazine*.

[bibr24-10944281241259075] Duşa A. (2018). QCA with R: A comprehensive resource. Springer.

[bibr25-10944281241259075] Elzinga C. H. Studer M. (2015). Spell sequences, state proximities, and distance metrics. Sociological Methods & Research, 44(1), 3–47. 10.1177/0049124114540707

[bibr26-10944281241259075] Fainshmidt S. Witt M. A. Aguilera R. V. Verbeke A. (2020). The contributions of qualitative comparative analysis (QCA) to international business research (Vol. 51, pp. 455–466). Springer.

[bibr27-10944281241259075] Fischer M. Maggetti M. (2017). Qualitative comparative analysis and the study of policy processes. Journal of Comparative Policy Analysis, 19(4), 345–361. 10.1080/13876988.2016.1149281

[bibr28-10944281241259075] Fiss P. (2011). Building better causal theories: A fuzzy set approach to typologies in organization research. Academy of Management Journal, 54(2), 393–420. 10.5465/amj.2011.60263120

[bibr29-10944281241259075] Fiss P. C. Zajac E. J. (2004). The diffusion of ideas over contested terrain: The (non)adoption of a shareholder value orientation among German firms. Administrative Science Quarterly, 49(4), 501–534. 10.2307/4131489

[bibr30-10944281241259075] Flanagan K. Uyarra E. Laranja M. (2011). Reconceptualising the ‘policy mix’ for innovation. Research Policy, 40(5), 702–713. 10.1016/j.respol.2011.02.005

[bibr31-10944281241259075] Fligstein N. Goldstein A. (2015). The emergence of a finance culture in American households, 1989–2007. Socio-Economic Review, 13(3), 575–601. 10.1093/ser/mwu035

[bibr32-10944281241259075] Furnari S. Crilly D. Misangyi V. F. Greckhamer T. Fiss P. C. Aguilera R. V. (2021). Capturing causal complexity: Heuristics for configurational theorizing. Academy of Management Review, 46(4), 778–799. 10.5465/amr.2019.0298

[bibr33-10944281241259075] Gabriel A. S. Campbell J. T. Djurdjevic E. Johnson R. E. Rosen C. (2018). Fuzzy profiles: Comparing and contrasting latent profile analysis and fuzzy set qualitative comparative analysis for person-centered research. Organizational Research Methods, 21(4), 877–904. 10.1177/1094428117752466

[bibr34-10944281241259075] García Castro R. Ariño Martín M. A. (2013). A general approach to panel data set-theoretic research. *COMPASSS WP Series 2013-76*.

[bibr35-10944281241259075] Gauthier J.-A. Widmer E. D. Bucher P. Notredame C. (2010). Multichannel sequence analysis applied to social science data. Sociological Methodology, 40(1), 1–38. 10.1111/j.1467-9531.2010.01227.x.

[bibr36-10944281241259075] Greckhamer T. (2011). Cross-cultural differences in compensation level and inequality across occupations: A set-theoretic analysis. Organization Studies, 32(1), 85–115. 10.1177/0170840610380806

[bibr37-10944281241259075] Greckhamer T. (2016). CEO compensation in relation to worker compensation across countries: The configurational impact of country-level institutions. Strategic Management Journal, 37(4), 793–815. 10.1002/smj.2370

[bibr38-10944281241259075] Greckhamer T. Furnari S. Fiss P. C. Aguilera R. V. (2018). Studying configurations with qualitative comparative analysis: Best practices in strategy and organization research. Strategic Organization, 16(4), 482–495. 10.1177/1476127018786487

[bibr39-10944281241259075] Greckhamer T. Misangyi V. Elms H. Lacey R. (2008). Using qualitative comparative analysis in strategic management research: An examination of combinations of industry, corporate, and business-unit effects. Organizational Research Methods, 11(4), 695–726. 10.1177/1094428107302907

[bibr40-10944281241259075] Greckhamer T. Misangyi V. Fiss P. (2013). The two QCAs: From a small to a large-N set theoretic approach. In Fiss P. Cambre B. Marx A. (Eds.), Configurational theory and methods in organizational research (pp. 49–75). Emerald Publishing.

[bibr41-10944281241259075] Gupta K. Crilly D. Greckhamer T. (2020). Stakeholder engagement strategies, national institutions, and firm performance: A configurational perspective. Strategic Management Journal, 41(10), 1869–1900. 10.1002/smj.3204

[bibr42-10944281241259075] Hak T. Jaspers F. Dul J. (2013). The analysis of temporally ordered configurations: Challenges and solutions. In Configurational theory and methods in organizational research. Emerald Group Publishing Limited.

[bibr43-10944281241259075] Halme M. Rintamäki J. Knudsen J. S. Lankoski L. Kuisma M. (2020). When is there a sustainability case for CSR? Pathways to environmental and social performance improvements. Business & Society, 59(6), 1181–1227. 10.1177/0007650318755648

[bibr44-10944281241259075] Hansmann H. Kraakman R. (2017). The end of history for corporate law. In Corporate governance (pp. 49–78). Gower.

[bibr45-10944281241259075] Hillman A. J. Keim G. D. (2001). Shareholder value, stakeholder management, and social issues: What’s the bottom line? Strategic Management Journal, 22(2), 125–139. 10.1002/1097-0266(200101)22:2<125::AID-SMJ150>3.0.CO;2-H

[bibr46-10944281241259075] Jeong Y.-C. Leblebici H. (2019). How professionalization and organizational diversity shape contemporary careers: Developing a typology and process model. Human Relations, 72(2), 298–321. 10.1177/0018726718761552

[bibr47-10944281241259075] Ketchen D. (2013). We try harder: Some reflections on configurational theory and methods. In Fiss Peer C. Cambré Bart Marx Axel (Eds.), Configurational theory and methods in organizational research (pp. 303–309). Emerald Group Publishing Limited.

[bibr48-10944281241259075] Koch M. Forgues B. Monties V. (2017). The way to the top: Career patterns of Fortune 100 CEOs. Human Resource Management, 56(2), 267–285. 10.1002/hrm.21759

[bibr49-10944281241259075] Koch M. Park S. Zahra S. A. (2021). Career patterns in self-employment and career success. Journal of Business Venturing, 36(1), 105998. 10.1016/j.jbusvent.2019.105998

[bibr50-10944281241259075] Langley A. Smallman C. Tsoukas H. Van de Ven A. H. (2013). Process studies of change in organization and management: Unveiling temporality, activity, and flow. Academy of Management Journal, 56(1), 1–13. 10.5465/amj.2013.4001

[bibr51-10944281241259075] La Porta R. Lopez-de-Silanes F. Shleifer A. (1999). Corporate ownership around the world. The Journal of Finance, 54(2), 471–517. 10.1111/0022-1082.00115

[bibr52-10944281241259075] Le Roux G. Studer M. Bringé A. Bonvalet C. (2020). Selecting qualitative cases using sequence analysis: A mixed-method strategy for in-depth understanding of life course trajectories. Advances in Life Course Research, 56, 100530. 10.1016/j.alcr.2023.100530 38054879

[bibr53-10944281241259075] Liao T. F. Bolano D. Brzinsky-Fay C. Cornwell B. Fasang A. E. Helske S. Piccarreta R. Raab M. Ritschard G. Struffolino E. Studer M. (2022). Sequence analysis: Its past, present, and future. Social Science Research, 107, 102772. 10.1016/j.ssresearch.2022.102772 36058612

[bibr54-10944281241259075] MacDuffie J. P. (1995). Human resource bundles and manufacturing performance: Organizational logic and flexible production systems in the world auto industry. Industrial and Labor Relations Review, 48(2), 197–221. 10.1177/001979399504800201

[bibr55-10944281241259075] Meuer J. (2017). Exploring the complementarities within high-performance work systems: A set-theoretic analysis of UK firms. Human Resource Management, 56(4), 651–672. 10.1002/hrm.21793

[bibr56-10944281241259075] Meuer J. Rupietta C. (2017a). Integrating QCA and HLM for multilevel research on organizational configurations. Organizational Research Methods, 20(2), 324–342. 10.1177/1094428116665465

[bibr57-10944281241259075] Meuer J. Rupietta C. (2017b). A review of integrated QCA and statistical analyses. Quality & Quantity, 51(5), 2063–2083. 10.1007/s11135-016-0397-z

[bibr58-10944281241259075] Meuer J. Rupietta C. Backes-Gellner U. (2015). Layers of co-existing innovation systems. Research Policy, 44(4), 888–910. 10.1016/j.respol.2015.01.013

[bibr59-10944281241259075] Meyer R. E. Höllerer M. A. (2010). Meaning structures in a contested issue field: A topographic map of shareholder value in Austria. Academy of Management Journal, 53(6), 1241–1262. 10.5465/amj.2010.57317829

[bibr60-10944281241259075] Miles R. H. Snow C. C. (1984). Designing strategic human resources systems. Organizational Dynamics, 13(1), 36–52. 10.1016/0090-2616(84)90030-5

[bibr61-10944281241259075] Miller D. (1987). The genesis of configuration. Academy of Management Review, 12(4), 686–701. 10.2307/258073

[bibr62-10944281241259075] Miller D. (1996). Configurations revisited. Strategic Management Journal, 17(7), 505–512. 10.1002/(SICI)1097-0266(199607)17:7<505::AID-SMJ852>3.0.CO;2-I

[bibr63-10944281241259075] Misangyi V. Acharya A. (2014). Substitutes or complements? A configurational examination of corporate governance mechanisms. Academy of Management Journal, 57(6), 1681–1705. 10.5465/amj.2012.0728

[bibr64-10944281241259075] Misangyi V. Greckhamer T. Furnari S. Fiss P. C. Crilly D. Aguilera R. (2017). Embracing causal complexity the emergence of a neo-configurational perspective. Journal of Management, 43(1), 255–282. 10.1177/0149206316679252

[bibr65-10944281241259075] Monge P. R. (1990). Theoretical and analytical issues in studying organizational processes. Organization Science, 1(4), 406–430. 10.1287/orsc.1.4.406

[bibr66-10944281241259075] Murray A. I. (1988). A contingency view of Porter’s “generic strategies”. Academy of Management Review, 13(3), 390–400. 10.2307/258087

[bibr67-10944281241259075] Nikou S. Mezei J. Liguori E. W. El Tarabishy A. (2022). FsQCA in entrepreneurship research: Opportunities and best practices. Journal of Small Business Management, 62(3), 1531–1548. 10.1080/00472778.2022.2147190

[bibr68-10944281241259075] Nishant R. Ravishankar M. (2020). QCA and the harnessing of unstructured qualitative data. Information Systems Journal, 30(5), 845–865. 10.1111/isj.12281

[bibr69-10944281241259075] Okoye A. (2009). Theorising corporate social responsibility as an essentially contested concept: Is a definition necessary? Journal of Business Ethics, 89(4), 613–627. 10.1007/s10551-008-0021-9

[bibr70-10944281241259075] Pagliarin S. Gerrits L. (2020). Trajectory-based qualitative comparative analysis: Accounting for case-based time dynamics. Methodological Innovations, 13(3). 10.1177/2059799120959170

[bibr71-10944281241259075] Pentland B. T. Mahringer C. A. Dittrich K. Feldman M. S. Wolf J. R. (2020). Process multiplicity and process dynamics: Weaving the space of possible paths. Organization Theory, 1(3). 10.1177/2631787720963138

[bibr72-10944281241259075] Prahalad C. K. (1994). Corporate governance or corporate value added?: Rethinking the primacy of shareholder value. Journal of Applied Corporate Finance, 6(4), 40–50. 10.1111/j.1745-6622.1994.tb00247.x

[bibr73-10944281241259075] Ragin C. (1987). The comparative method: Moving beyond qualitative and quantitative strategies. University of California Press.

[bibr74-10944281241259075] Ragin C. (1994). A qualitative comparative analysis of pension systems. In Janoski T. Hicks A. M. (Eds.), The comparative political economy of the welfare state (pp. 300–309). Cambridge University Press.

[bibr75-10944281241259075] Ragin C. (2000). Fuzzy-set social science. University of Chicago Press.

[bibr76-10944281241259075] Ragin C. (2008). Redesigning social inquiry. University of Chicago Press.

[bibr77-10944281241259075] Ragin C. Davey S. (2016). Fuzzy-set/qualitative comparative analysis 3.0. Department of Sociology, University of California.

[bibr78-10944281241259075] Raisch S. Hargrave T. J. Van De Ven A. H. (2018). The learning spiral: A process perspective on paradox. Journal of Management Studies, 55(8), 1507–1526. 10.1111/joms.12397

[bibr79-10944281241259075] Rappaport A. (2006). Ways to create shareholder value. Harvard Business Review, 84(9), 66–77. 16967621

[bibr80-10944281241259075] Ritschard G. Studer M. (2018). Sequence analysis: Where are we, where are we going? In Ritschard G. Studer M. (Eds.), Sequence analysis and related approaches: Innovative methods and applications (pp. 1–11). SpringerOpen.

[bibr81-10944281241259075] Rogge K. S. Reichardt K. (2016). Policy mixes for sustainability transitions: An extended concept and framework for analysis. Research Policy, 45(8), 1620–1635. 10.1016/j.respol.2016.04.004

[bibr82-10944281241259075] Rubach M. J. Sebora T. C. (1998). Comparative corporate governance: Competitive implications of an emerging convergence. Journal of World Business, 33(2), 167–184. 10.1016/S1090-9516(98)90004-9

[bibr83-10944281241259075] Rubinson C. Gerrits L. Rutten R. Greckhamer T. . (2019). *Avoiding common errors in QCA: A short guide for new practitioners* (pp. 1–6). COMPASSS Research Network.

[bibr84-10944281241259075] Schneider C. Q. (2019). Two-step QCA revisited: The necessity of context conditions. Quality & Quantity, 53(3), 1109–1126. 10.1007/s11135-018-0805-7

[bibr85-10944281241259075] Schneider C. Q. Rohlfing I. (2013). Combining QCA and process tracing in set-theoretic multi-method research. Sociological Methods & Research, 42(4), 559–597. 10.1177/0049124113481341

[bibr86-10944281241259075] Schneider C. Q. Rohlfing I. (2016). Case studies nested in fuzzy-set QCA on sufficiency: Formalizing case selection and causal inference. Sociological Methods & Research, 45(3), 526–568. 10.1177/0049124114532446

[bibr87-10944281241259075] Schneider C. Q. Wagemann C. (2013). Set-theoretic methods for the social sciences: A guide to qualitative comparative analysis. Cambridge University Press.

[bibr88-10944281241259075] Seo H.-J. Kim H. S. Kim J. (2016). Does shareholder value orientation or financial market liberalization slow down Korean real investment? Review of Radical Political Economics, 48(4), 633–660. 10.1177/0486613415603159

[bibr89-10944281241259075] Short J. C. Payne G. T. Ketchen D. J. (2008). Research on organizational configurations: Past accomplishments and future challenges. Journal of Management, 34(6), 1053–1079. 10.1177/0149206308324324

[bibr90-10944281241259075] Slager R. Chuah K. Gond J.-P. Furnari S. Homanen M. (2023). Tailor-to-target: Configuring collaborative shareholder engagements on climate change. Management Science, 69(12), 7693–7718. 10.1287/mnsc.2023.4806

[bibr91-10944281241259075] Storz C. (2008). Dynamics in innovation systems: Evidence from Japan’s game software industry. Research Policy, 37(9), 1480–1491. 10.1016/j.respol.2008.05.007

[bibr92-10944281241259075] Stout L. A. (2012). New thinking on “shareholder primacy”. Accounting, Economics, and Law, 2(2), 2–21. 10.1515/2152-2820.1037

[bibr93-10944281241259075] Studer M. Ritschard G. (2016). What matters in differences between life trajectories: A comparative review of sequence dissimilarity measures. Journal of the Royal Statistical Society. Series A (Statistics in Society), 179(2), 481–511. 10.1111/rssa.12125

[bibr94-10944281241259075] Tashakkori A. Teddlie C. (2008). Quality of inferences in mixed methods research: Calling for an integrative framework. In Bergmann M. (Ed.), Advances in mixed methods research (pp. 101–119). SAGE.

[bibr95-10944281241259075] Van de Ven A. H. (2007). Engaged scholarship: A guide for organizational and social research. Oxford University Press.

[bibr96-10944281241259075] Van de Ven A. H. Poole M. S. (1995). Explaining development and change in organizations. Academy of Management Review, 20(3), 510–540. 10.2307/258786

[bibr97-10944281241259075] Venkatraman N. Loh L. Koh J. (1994). The adoption of corporate governance mechanisms: A test of competing diffusion models. Management Science, 40(4), 496–507. 10.1287/mnsc.40.4.496

[bibr98-10944281241259075] Verweij S. Vis B. (2021). Three strategies to track configurations over time with qualitative comparative analysis. European Political Science Review, 13(1), 95–111. 10.1017/S1755773920000375

[bibr99-10944281241259075] Zajac E. J. Shortell S. M. (1989). Changing generic strategies: Likelihood, direction, and performance implications. Strategic Management Journal, 10(5), 413–430. 10.1002/smj.4250100503

[bibr100-10944281241259075] Zimmermann A. Raisch S. Cardinal L. B. (2018). Managing persistent tensions on the frontline: A configurational perspective on ambidexterity. Journal of Management Studies, 55(5), 739–769. 10.1111/joms.12311

