# Supplementary material for: Comparative Configurational Process Analysis: A New Set-Theoretic Technique for Longitudinal Case Analysis
Source: Organ Res Methods. 2024 Jun 18;28(3):405–32. doi: 10.1177/10944281241259075 (PMC12225975; doi:10.1177/10944281241259075)
Supplement: sj-docx-1-orm-10.1177_10944281241259075 - Supplemental material for Comparative Configurational Process Analysis: A New Set-Theoretic Technique for Longitudinal Case Analysis [file sj-docx-1-orm-10.1177_10944281241259075.docx]

# Comparative configurational process analysis (C^2^PA): A new set-theoretic technique for longitudinal case analysis

# Supplementary File A Scale, items, and descriptive statistics for the outcome measure of shareholder value orientation

The outcome measure for our analyses is a scale that combines several aspects of shareholder value orientation (SVO) and allows the generation of a balanced panel with properties that align with the data requirements of QCA and sequence analysis. The generation of the outcome measure follows four steps: First, we identify suitable SVO measures; second, we prepare the data to obtain a balanced panel data; third, we calculate the scale; fourth, the scale is calibrated. Steps two and three work in an iterative fashion because the outcome measure determines not only the size of the panel dataset but also the data availability for the conditions (i.e., the shares held by different investor groups) in our QCA model.

In the first step, we use the Thomson Reuters database to obtain data on the SVO of all the companies that have been listed at least once in the HDAX between 2003 and 2018. The HDAX combines the DAX, MDAX, and TecDAX and, thus, covers the 110 largest German companies. We identified 48 SVO measures in the database. Table A.1 shows the descriptive statistics of the available SVO measures from 2003–2018. As Table A. 1 shows several items contain many missing values or have little variation. To identify measures that allow us to discriminate between companies’ adoption of SV, we exclude measures with missing values and no, or only a little, variation.

In the second step, we analyze the structure of the missing values in the available SVO measures and detect data availability problems, especially for the years 2003–2005 and 2018. After excluding SVO measures that were non-binary and had no variation and/or few observations, as the third step, we developed an initial SVO scale that represented the yearly average of the available SVO measures. We repeated these steps after linking the dates to the conditions to develop our final scale measuring a firm’s SVO. Table A.2 shows the descriptive statistics of the nine SVO measures that form the SVO scale for the period of 2006–2017.

In the fourth step, we calibrated the SVO scale to generate the outcome for the QCA model. Given the nature of our SVO scale, we use the direct calibration drawing on the distribution of the measures. In calibrating the scale, we paid particular attention to incorporating general trends in the development of the SVO over the period of 2006–2017. We thus defined yearly anchors points based on the entire population of companies listed in the HDAX. Specifically, we defined the 10^th^ percentile as the threshold for being fully out of the set, the mean as the cross-over point, and the 90^th^ percentile as the threshold for being fully in the set. With these calibration points, we thus define the set of companies with above-average annual SVO. The selected anchors were meaningful because they built on an approximation of a representative population (i.e., all companies in the HDAX) and classified the companies that beat the SVO average of this population as having a high SVO. Table A.3 shows the calibrated and uncalibrated measures for the outcome and conditions. The outcome reflects—with a mean close to 0.5—the definition of the yearly SVO mean in the HDAX as the point of maximum ambiguity.

Table A.1: Descriptive statistics on Shareholder Value Orientation Measures for all companies listed in the HDAX from 2003-2018

| Variable | Obs | Mean | Std. Dev. | Min | Max |
| --- | --- | --- | --- | --- | --- |
| Shareholder Rights Policy | 1,252 | 0.938 | 0.242 | 0 | 1 |
| Policy Equal Voting Right | 1,252 | 0.932 | 0.252 | 0 | 1 |
| Policy Shareholder Engagement | 1,252 | 0.538 | 0.499 | 0 | 1 |
| Different Voting Right Share | 1,252 | 0.060 | 0.237 | 0 | 1 |
| Equal Shareholder Rights | 1,252 | 0.934 | 0.249 | 0 | 1 |
| Voting Cap | 1,252 | 0.002 | 0.040 | 0 | 1 |
| Voting Cap Percentage | 1,114 | 100.000 | 0.000 | 100 | 100 |
| Minimum Number of Shares to Vote | 1,252 | 0.002 | 0.049 | 0 | 1 |
| Director Election Majority Requirement | 1,252 | 0.599 | 0.490 | 0 | 1 |
| Shareholders Vote on Executive Pay | 1,252 | 0.256 | 0.437 | 0 | 1 |
| Public Availability Corporate Statutes | 1,078 | 0.994 | 0.074 | 0 | 1 |
| Veto Power or Golden share | 1,152 | 0.179 | 0.383 | 0 | 1 |
| State Owned Enterprise SOE | 503 | 0.006 | 0.077 | 0 | 1 |
| Anti Takeover Devices Above Two | 1,253 | 1.972 | 1.714 | 0 | 9 |
| Poison Pill | 319 | 0.000 | 0.000 | 0 | 0 |
| Unlimited Authorized Capital or Blank Check | 1,191 | 0.013 | 0.112 | 0 | 1 |
| Classified Board Structure | 1,188 | 0.096 | 0.295 | 0 | 1 |
| Staggered Board Structure | 1,134 | 0.250 | 0.433 | 0 | 1 |
| Supermajority Vote Requirement | 1,132 | 0.655 | 0.476 | 0 | 1 |
| Golden Parachute | 1,121 | 0.452 | 0.498 | 0 | 1 |
| Limited Shareholder Rights to Call Meetings | 814 | 0.536 | 0.499 | 0 | 1 |
| Elimination of Cumulative Voting Rights | 330 | 0.106 | 0.308 | 0 | 1 |
| Pre-emptive Rights | 1,184 | 0.834 | 0.373 | 0 | 1 |
| Company Cross Shareholding | 338 | 0.003 | 0.054 | 0 | 1 |
| Confidential Voting Policy | 334 | 0.024 | 0.153 | 0 | 1 |
| Limitation of Director Liability | 1,064 | 0.729 | 0.445 | 0 | 1 |
| Shareholder Rights Controversies Count | 21 | 1.000 | 0.000 | 1 | 1 |
| Recent Shareholder Rights Controversies | 2 | 1.000 | 0.000 | 1 | 1 |
| Shareholder Approval Significant Transactions | 27 | 0.704 | 0.465 | 0 | 1 |
| Fair Price Provision | 4 | 1.000 | 0.000 | 1 | 1 |
| Limitations on Removal of Directors | 130 | 0.754 | 0.432 | 0 | 1 |
| Advance Notice for Shareholder Proposals | 751 | 0.992 | 0.089 | 0 | 1 |
| Advance Notice Period Days | 418 | 30.200 | 10.450 | 7 | 90 |
| Written Consent Requirements | 8 | 0.250 | 0.463 | 0 | 1 |
| Earnings Restatement | 1,252 | 0.023 | 0.150 | 0 | 1 |
| Profit Warnings | 1,252 | 0.175 | 0.380 | 0 | 1 |
| Litigation Expenses | 213 | 1.180e+08 | 5.490e+08 | -7.170e+08 | 5.100e+09 |
| Non-audit to Audit Fees Ratio | 1,098 | 0.337 | 0.597 | 0 | 9.671 |
| Auditor Independence Rotation | 1,149 | 4.245 | 2.437 | 1 | 15 |
| Insider Dealings Controversies | 1,252 | 0.023 | 0.150 | 0 | 1 |
| Insider Dealings Controversies Count | 35 | 0.943 | 0.338 | 0 | 2 |
| Recent Insider Dealings Controversies | 2 | 1.000 | 0.000 | 1 | 1 |
| Accounting Controversies | 1,252 | 0.010 | 0.101 | 0 | 1 |
| Accounting Controversies Count | 15 | 0.933 | 0.458 | 0 | 2 |
| Recent Accounting Controversies Count | 3 | 4.333 | 5.774 | 1 | 11 |

Table A.2: Descriptive statistics on Shareholder Value Orientation Measures that form the Shareholder Value Orientation Scale (Balanced Panel, 2006-2017)

| Variable | Obs | Mean | Std. Dev. | Min | Max |
| --- | --- | --- | --- | --- | --- |
| Shareholder Rights Policy | 648 | 0.981 | 0.135 | 0 | 1 |
| Policy Shareholder Engagement | 648 | 0.619 | 0.486 | 0 | 1 |
| Shareholders Vote on Executive Pay | 648 | 0.318 | 0.466 | 0 | 1 |
| Unlimited Authorized Capital or Blank Check | 617 | 0.016 | 0.126 | 0 | 1 |
| Elimination of Cumulative Voting Rights | 143 | 0.224 | 0.418 | 0 | 1 |
| Pre-emptive Rights | 638 | 0.898 | 0.303 | 0 | 1 |
| Limitations on Removal of Directors | 104 | 0.769 | 0.423 | 0 | 1 |
| Supermajority Vote Requirement (rev) | 599 | 0.275 | 0.447 | 0 | 1 |
| Limited Shareholder Rights to Call Meetings (rev) | 447 | 0.407 | 0.492 | 0 | 1 |

Table A.3 Calibrated and uncalibrated outcome and conditions for the QCA model.

| Outcome and Conditions | Obs | Mean | Std. Dev. | Min | Max |
| --- | --- | --- | --- | --- | --- |
| **Calibrated** |  |  |  |  |  |
| **Outcome** |  |  |  |  |  |
| *The set of companies with above-average annual SVO* | 648 | 0.564 | 0.345 | 0.001 | 1.000 |
| **Conditions** |  |  |  |  |  |
| *The set of companies with influential….* |  |  |  |  |  |
| Hedge Funds | 648 | 0.013 | 0.077 | 0.001 | 0.781 |
| Government | 648 | 0.056 | 0.208 | 0.001 | 0.961 |
| Corporation | 648 | 0.291 | 0.415 | 0.001 | 1.000 |
| Holding | 648 | 0.084 | 0.260 | 0.001 | 1.000 |
| Indiv. Investor | 648 | 0.136 | 0.308 | 0.001 | 1.000 |
|  |  |  |  |  |  |
| **Uncalibrated** |  |  |  |  |  |
| **Outcome** |  |  |  |  |  |
| Shareholder Value Orientation | 648 | 0.511 | 0.144 | 0 | 0.857 |
| **Conditions** |  |  |  |  |  |
| Percentage of shares held by |  |  |  |  |  |
| Hedge Funds | 648 | 0.703 | 1.135 | 0.026 | 13.460 |
| Government | 648 | 1.201 | 4.737 | 0 | 26.500 |
| Corporation | 648 | 9.458 | 17.860 | 0 | 85.810 |
| Holding | 648 | 3.072 | 11.150 | 0 | 65.000 |
| Indiv. Investor | 648 | 3.411 | 9.148 | 0 | 49.900 |
