# Supplementary material for: Comparative Configurational Process Analysis: A New Set-Theoretic Technique for Longitudinal Case Analysis
Source: Organ Res Methods. 2024 Jun 18;28(3):405–32. doi: 10.1177/10944281241259075 (PMC12225975; doi:10.1177/10944281241259075)
Supplement: sj-docx-2-orm-10.1177_10944281241259075 - Supplemental material for Comparative Configurational Process Analysis: A New Set-Theoretic Technique for Longitudinal Case Analysis [file sj-docx-2-orm-10.1177_10944281241259075.docx]

# Comparative configurational process analysis (C^2^PA): A new set-theoretic technique for longitudinal case analysis

# Supplementary File B Aggregated yearly truth tables

This supplementary file contains the yearly truth tables in an aggregated format. The first column numbers each of the 32 possible configurations. The conditions for each investor type are listed with 0 or 1, indicating the presence or absence of a high percentage of shares held by an investor type. The cells show the frequency of cases as well as the raw consistency and, in parenthesis, the PRI consistency. All values are rounded to the second digit. We used unrounded consistency values and a consistency threshold of 0.80 (0.70 for PRI) to select configurations for the Boolean Minimization. The numbers in bold indicate those truth table rows that we selected for the minimization.

## Table B.1 Aggregated truth table (from the cross 2006-2017) for the set of firms with an above-average annual SVO.

| # | Hedge Funds | Government | Corporation | Holding | Individual Investor | 2006 | 2007 | 2008 | 2009 | 2010 | 2011 | 2012 | 2013 | 2014 | 2015 | 2016 | 2017 |
| --- | --- | --- | --- | --- | --- | --- | --- | --- | --- | --- | --- | --- | --- | --- | --- | --- | --- |
| 1 | 0 | 0 | 0 | 0 | 0 | **28; 0.74 (0.73)** | 26; 0.63 (0.53) | 23; 0.60 (0.45) | 24; 0.51 (0.35) | 23; 0.57 (0.43) | 24; 0.66 (0.54) | 23; 0.57 (0.42) | 24; 0.59 (0.44) | 23; 0.60 (0.47) | 26; 0.64 (0.50) | 25; 0.61 (0.48) | 24; 0.59 (0.47) |
| 2 | 0 | 0 | 0 | 0 | 1 | **4; 0.84 (0.82)** | **2; 0.92 (0.87)** | **4; 0.95 (0.93)** | **3; 0.99 (0.98)** | 5; 0.75 (0.68) | 5; 0.79 (0.68) | 6; 0.77 (0.64) | **6; 0.80 (0.66)** | **9; 0.83 (0.75)** | **7; 0.80 (0.71)** | **8; 0.83 (0.77)** | 7; 0.64 (0.49) |
| 3 | 0 | 0 | 0 | 1 | 0 | 4; 0.49 (0.44) | 4; 0.45 (0.28) | 3; 0.45 (0.34) | 4; 0.57 (0.35) | 3; 0.58 (0.43) | 4; 0.48 (0.29) | 3; 0.63 (0.51) | **2; 0.79 (0.73)** | **2; 0.91 (0.89)** | **2; 0.82 (0.79)** | **2; 0.83 (0.80)** | **3; 0.80 (0.77)** |
| 4 | 0 | 0 | 0 | 1 | 1 | 0; - (-) | **1; 1.00 (1.00)** | 0; - (-) | 0; - (-) | 0; - (-) | 0; - (-) | 0; - (-) | 1; 0.38 (0.00) | 1; 0.39 (0.00) | 1; 0.14 (0.00) | 1; 0.20 (0.07) | 1; 0.16 (0.07) |
| 5 | 0 | 0 | 1 | 0 | 0 | 11; 0.60 (0.57) | 13; 0.44 (0.29) | 13; 0.60 (0.48) | 12; 0.43 (0.18) | 13; 0.44 (0.26) | 12; 0.42 (0.24) | 15; 0.56 (0.44) | 13; 0.55 (0.43) | 13; 0.51 (0.41) | 12; 0.52 (0.38) | 12; 0.55 (0.46) | 11; 0.58 (0.49) |
| 6 | 0 | 0 | 1 | 0 | 1 | **1; 1.00 (1.00)** | 2; 0.62 (0.44) | 4; 0.72 (0.59) | **4; 0.88 (0.81)** | **3; 1.00 (1.00)** | **3; 0.96 (0.90)** | **2; 0.91 (0.80)** | **3; 0.96 (0.94)** | 2; 0.70 (0.63) | 2; 0.78 (0.69) | 2; 0.70 (0.63) | **3; 0.92 (0.88)** |
| 7 | 0 | 0 | 1 | 1 | 0 | 2; 0.17 (0.01) | 2; 0.62 (0.43) | 2; 0.71 (0.61) | 2; 0.82 (0.43) | 2; 0.68 (0.37) | 1; 0.53 (0.00) | **1; 1.00 (1.00)** | 0; - (-) | 0; - (-) | 0; - (-) | 0; - (-) | 0; - (-) |
| 8 | 0 | 0 | 1 | 1 | 1 | 0; - (-) | 0; - (-) | 0; - (-) | **1; 1.00 (1.00)** | **1; 1.00 (1.00)** | **1; 1.00 (1.00)** | **1; 1.00 (1.00)** | 0; - (-) | 0; - (-) | 0; - (-) | 0; - (-) | 0; - (-) |
| 9 | 0 | 1 | 0 | 0 | 0 | **1; 1.00 (1.00)** | 1; 0.44 (0.07) | 1; 0.37 (0.01) | 3; 0.64 (0.56) | 3; 0.79 (0.65) | 3; 0.69 (0.52) | 2; 0.71 (0.59) | 3; 0.69 (0.60) | 3; 0.70 (0.61) | 3; 0.73 (0.62) | **3; 0.82 (0.74)** | 2; 0.75 (0.61) |
| 10 | 0 | 1 | 0 | 0 | 1 | 0; - (-) | 0; - (-) | 0; - (-) | 0; - (-) | 0; - (-) | 0; - (-) | 0; - (-) | 0; - (-) | 0; - (-) | 0; - (-) | 0; - (-) | 0; - (-) |
| 11 | 0 | 1 | 0 | 1 | 0 | 0; - (-) | 0; - (-) | 1; 0.18 (0.01) | 0; - (-) | 0; - (-) | 0; - (-) | 0; - (-) | 0; - (-) | 0; - (-) | 0; - (-) | 0; - (-) | 0; - (-) |
| 12 | 0 | 1 | 0 | 1 | 1 | 0; - (-) | 0; - (-) | 0; - (-) | 0; - (-) | 0; - (-) | 0; - (-) | 0; - (-) | 0; - (-) | 0; - (-) | 0; - (-) | 0; - (-) | 0; - (-) |
| 13 | 0 | 1 | 1 | 0 | 0 | **2; 1.00 (1.00)** | **1; 0.98 (0.96)** | 1; 0.79 (0.60) | **1; 0.90 (0.86)** | 1; 0.44 (0.28) | 1; 0.19 (0.00) | 1; 0.11 (0.00) | 1; 0.12 (0.00) | 1; 0.12 (0.00) | 1; 0.17 (0.00) | 1; 0.16 (0.01) | 1; 0.09 (0.01) |
| 14 | 0 | 1 | 1 | 0 | 1 | 0; - (-) | 0; - (-) | 0; - (-) | 0; - (-) | 0; - (-) | 0; - (-) | 0; - (-) | 0; - (-) | 0; - (-) | 0; - (-) | 0; - (-) | 0; - (-) |
| 15 | 0 | 1 | 1 | 1 | 0 | 0; - (-) | 0; - (-) | 0; - (-) | 0; - (-) | 0; - (-) | 0; - (-) | 0; - (-) | 0; - (-) | 0; - (-) | 0; - (-) | 0; - (-) | 0; - (-) |
| 16 | 0 | 1 | 1 | 1 | 1 | 0; - (-) | 0; - (-) | 0; - (-) | 0; - (-) | 0; - (-) | 0; - (-) | 0; - (-) | 0; - (-) | 0; - (-) | 0; - (-) | 0; - (-) | 0; - (-) |
| 17 | 1 | 0 | 0 | 0 | 0 | 1; 0.47 (0.39) | 2; 0.61 (0.46) | 2; 0.79 (0.72) | 0; - (-) | 0; - (-) | 0; - (-) | 0; - (-) | 0; - (-) | 0; - (-) | 0; - (-) | 0; - (-) | 0; - (-) |
| 18 | 1 | 0 | 0 | 0 | 1 | 0; - (-) | 0; - (-) | 0; - (-) | 0; - (-) | 0; - (-) | 0; - (-) | 0; - (-) | 0; - (-) | 0; - (-) | 0; - (-) | 0; - (-) | 0; - (-) |
| 19 | 1 | 0 | 0 | 1 | 0 | 0; - (-) | 0; - (-) | 0; - (-) | 0; - (-) | 0; - (-) | 0; - (-) | 0; - (-) | 1; 0.20 (0.00) | 0; - (-) | 0; - (-) | 0; - (-) | 1; 0.43 (0.01) |
| 20 | 1 | 0 | 0 | 1 | 1 | 0; - (-) | 0; - (-) | 0; - (-) | 0; - (-) | 0; - (-) | 0; - (-) | 0; - (-) | 0; - (-) | 0; - (-) | 0; - (-) | 0; - (-) | 0; - (-) |
| 21 | 1 | 0 | 1 | 0 | 0 | 0; - (-) | 0; - (-) | 0; - (-) | 0; - (-) | 0; - (-) | 0; - (-) | 0; - (-) | 0; - (-) | 0; - (-) | 0; - (-) | 0; - (-) | 0; - (-) |
| 22 | 1 | 0 | 1 | 0 | 1 | 0; - (-) | 0; - (-) | 0; - (-) | 0; - (-) | 0; - (-) | 0; - (-) | 0; - (-) | 0; - (-) | 0; - (-) | 0; - (-) | 0; - (-) | 0; - (-) |
| 23 | 1 | 0 | 1 | 1 | 0 | 0; - (-) | 0; - (-) | 0; - (-) | 0; - (-) | 0; - (-) | 0; - (-) | 0; - (-) | 0; - (-) | 0; - (-) | 0; - (-) | 0; - (-) | 0; - (-) |
| 24 | 1 | 0 | 1 | 1 | 1 | 0; - (-) | 0; - (-) | 0; - (-) | 0; - (-) | 0; - (-) | 0; - (-) | 0; - (-) | 0; - (-) | 0; - (-) | 0; - (-) | 0; - (-) | 0; - (-) |
| 25 | 1 | 1 | 0 | 0 | 0 | 0; - (-) | 0; - (-) | 0; - (-) | 0; - (-) | 0; - (-) | 0; - (-) | 0; - (-) | 0; - (-) | 0; - (-) | 0; - (-) | 0; - (-) | 1; 1.00 (1.00) |
| 26 | 1 | 1 | 0 | 0 | 1 | 0; - (-) | 0; - (-) | 0; - (-) | 0; - (-) | 0; - (-) | 0; - (-) | 0; - (-) | 0; - (-) | 0; - (-) | 0; - (-) | 0; - (-) | 0; - (-) |
| 27 | 1 | 1 | 0 | 1 | 0 | 0; - (-) | 0; - (-) | 0; - (-) | 0; - (-) | 0; - (-) | 0; - (-) | 0; - (-) | 0; - (-) | 0; - (-) | 0; - (-) | 0; - (-) | 0; - (-) |
| 28 | 1 | 1 | 0 | 1 | 1 | 0; - (-) | 0; - (-) | 0; - (-) | 0; - (-) | 0; - (-) | 0; - (-) | 0; - (-) | 0; - (-) | 0; - (-) | 0; - (-) | 0; - (-) | 0; - (-) |
| 29 | 1 | 1 | 1 | 0 | 0 | 0; - (-) | 0; - (-) | 0; - (-) | 0; - (-) | 0; - (-) | 0; - (-) | 0; - (-) | 0; - (-) | 0; - (-) | 0; - (-) | 0; - (-) | 0; - (-) |
| 30 | 1 | 1 | 1 | 0 | 1 | 0; - (-) | 0; - (-) | 0; - (-) | 0; - (-) | 0; - (-) | 0; - (-) | 0; - (-) | 0; - (-) | 0; - (-) | 0; - (-) | 0; - (-) | 0; - (-) |
| 31 | 1 | 1 | 1 | 1 | 0 | 0; - (-) | 0; - (-) | 0; - (-) | 0; - (-) | 0; - (-) | 0; - (-) | 0; - (-) | 0; - (-) | 0; - (-) | 0; - (-) | 0; - (-) | 0; - (-) |
| 32 | 1 | 1 | 1 | 1 | 1 | 0; - (-) | 0; - (-) | 0; - (-) | 0; - (-) | 0; - (-) | 0; - (-) | 0; - (-) | 0; - (-) | 0; - (-) | 0; - (-) | 0; - (-) | 0; - (-) |
