# Supplementary material for: Comparative Configurational Process Analysis: A New Set-Theoretic Technique for Longitudinal Case Analysis
Source: Organ Res Methods. 2024 Jun 18;28(3):405–32. doi: 10.1177/10944281241259075 (PMC12225975; doi:10.1177/10944281241259075)
Supplement: sj-docx-3-orm-10.1177_10944281241259075 - Supplemental material for Comparative Configurational Process Analysis: A New Set-Theoretic Technique for Longitudinal Case Analysis [file sj-docx-3-orm-10.1177_10944281241259075.docx]

Comparative configurational process analysis (C^2^PA):
A new set-theoretic technique for longitudinal case analysis

Supplementary File C **Configuration Charts for Weak SVO**


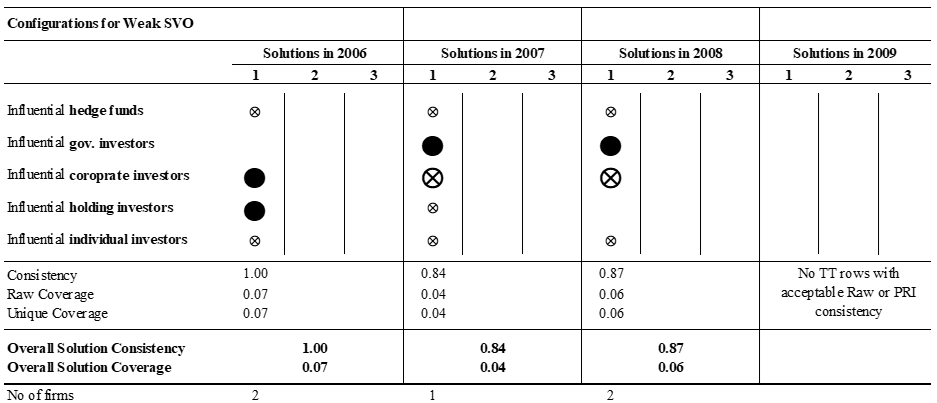


Figure C.1 Configuration charts 2006-2009.


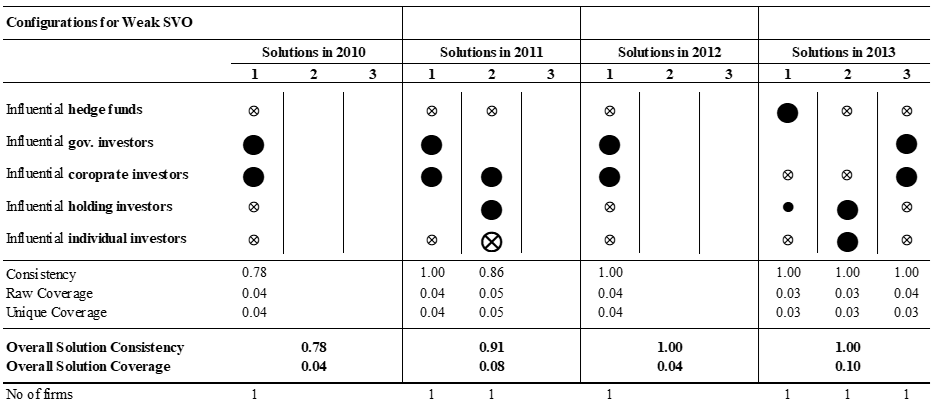


Figure C.2 Configuration charts 2010-2013.


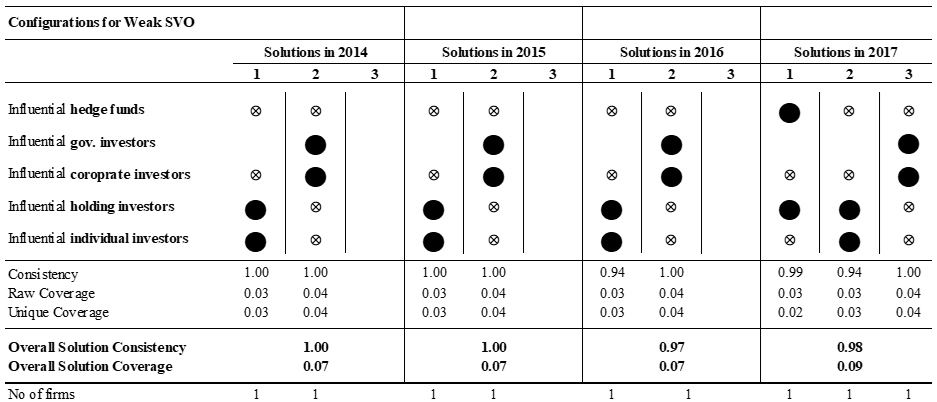


Figure C.3 Configuration charts 2014-2017.
